# Transepithelial Anti-Neuroblastoma Response to Kale among Four Vegetable Juices Using In Vitro Model Co-Culture System

**DOI:** 10.3390/nu13020488

**Published:** 2021-02-02

**Authors:** John E. Piletz, Yuhan Mao, Debarshi Roy, Bilal Qizilbash, Eurielle Nkamssi, Enleyona Weir, Jessica Graham, Mary Emmanuel, Suwaira Iqbal, Kellie Brue, Bidisha Sengupta

**Affiliations:** 1 Department of Biology, Mississippi College, Clinton, MS 39058, USA; rebecca.1990@163.com (Y.M.); egnjofangnkamssi@mc.edu (E.N.); savera.iqbal@gmail.com (S.I.); kellie.brue@med.wayne.edu (K.B.); 2 Department of Biology, Alcorn State University, Lorman, MS 39096, USA; droy@alcorn.edu; 3 Qizilbash Labs, 345 Woodstone Road, Suite K6, Clinton, MS 39056, USA; bilal@easykale.com; 4 Department of Chemistry, Tougaloo College, 500 West County Line Road, Tougaloo, MS 39174, USA; eweir42@gmail.com (E.W.); jessib3194@gmail.com (J.G.); amarachi87@yahoo.com (M.E.); 5 Department of Chemistry and Biochemistry, Stephen F. Austin State University, Nacogdoches, TX 75962, USA

**Keywords:** *Brassica oleracea*, cruciferous, *Taraxacum officinale*, *Spinacia oleracea*, *Lactuca sativa*, vegetable juice, *Lactobacillus fermentum*, *Caco-2*, *SH-SY5Y*, transepithelial electrical resistance, tight junctions, bioavailability, bio accessibility, kaempferol, oxalate

## Abstract

Juicing vegetables is thought to be an anticancer treatment. Support exists for a rank order of anticancer greens (kale > dandelion > lettuce > spinach) based on degrees of bioavailability of different phytochemicals, also offset by some noxious molecules (i.e., calcium-oxalate). We developed a new in vitro transepithelial anti-neuroblastoma model system. The juices were diluted as predicted once in the small intestine. They were applied to apical *Caco-2Bbe1* cells atop dividing *SH-SY5Y* neuroblastoma cells, and changes in transepithelial electrical resistance (TEER) and cell growth were considered with juice spectroscopies. Studied first in monoculture, kale and dandelion were the most cytostatic juices on *SH-SY5Y*s, lettuce showed no effect, and high (4.2%) spinach was cytotoxic. In co-culture, high (4.2%) kale was quickest (three days) to inhibit neuroblastoma growth. By five days, dandelion and kale were equally robust. Lettuce showed small anti-proliferative effects at five days and spinach remained cytotoxic. Spinach’s cytotoxicity corresponded with major infrared bands indicative of oxalate. Kale juice uniquely induced reactive oxygen species and S-phase cell cycle arrest in *SH-SY5Y*. The superiority of kale and dandelion was also apparent on the epithelium, because raising TEER levels is considered healthy. Kale’s unique features corresponded with a major fluorescent peak that co-eluted with kaempferol during high performance liquid chromatography. Because the anticancer rank order was upheld, the model appears validated for screening anticancer juices.

## 1. Introduction

**Scheme 1:**
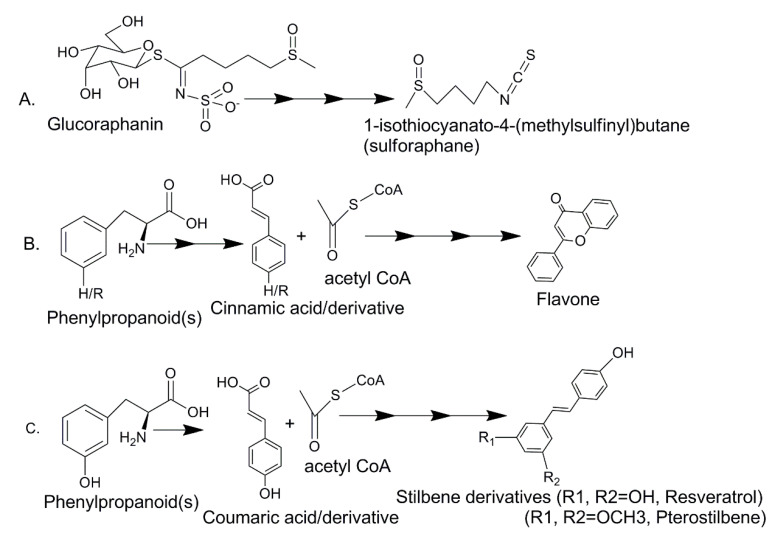
Chemical structures of the phytochemicals abundant in green curly kale: (**A**) Biosynthesis of sulforaphane from precursor glucoraphanin [45]; (**B**) Biosynthesis of flavone from precursor phenylpropanoids [7]; (**C**) Biosynthesis of the stilbenes, resveratrol and pterostilbene, also from precursor phenylpropanoids [13]. Multiple arrows indicate the occurrence of steps and intermediates during the biosynthesis.

Green smoothies and juices are popular, most often containing *Brassica* vegetables [1,2]. Not for being sweet like fruit, but because diets rich in cruciferous vegetables are associated with a low risk of breast, liver, lung, prostate, and cervical cancer [2,3,4,5,6,7,8,9], and probably many other cancers [10,11]. In particular, curly kale (*Brassica oleracea* L. var. *sabellica*, red or green), is called a superfood and anticancer [12]. This is thought to owe to kale’s abundant and diverse phytochemical flavonoids (Scheme 1B), also found in other *Brassica* such as cauliflower, broccoli, Brussel sprouts, and cabbages [6,13,14]. Yet, different phytochemicals exist in different plants [15] consistent with each plant’s need to defend itself against different pathogens, predators, and conditions [16]. Eating greens delivers a complex mixture of phytochemicals, be they chelating [17], antifungal [18], antiviral [19], antimutagen [8,20], antioxidant [21,22], anti-inflammatory [23], and/or anticancer [24,25]. Fermentation enhances some [26]. Cooking denatures most types [3]. By and large, most phytochemicals are best prepared for human consumption by fresh juicing [27].

The top three phytochemical anticancer pathways found in most green vegetables are shown in Scheme 1. They are the isothiocyanate, flavone/flavonoid, and stilbene pathways. Studies using these purified phytochemicals have revealed that many act against cancer in synergy with known chemotherapeutic mechanisms [28,29]. Based on their known safety and few (if any) adverse interactions, general practitioners may thus confidently prescribe phytochemical pills as preventatives (i.e., kaempferol pills) [7], and oncologists may consider phytochemicals for adjunct or adjuvant therapies [10]. Standard chemotherapies (i.e., cyclophosphamide, cisplatin, vincristine, doxorubicin [30]) are harsh on the gut [31] and more pills are sometimes shunned [32]. So, might a green juice be a better adjunct therapy than a phytochemical pill? At minimum, green smoothies and juices provide nutritional value [33]. Compliance is also a big problem for treating neuroblastoma where 90% of the cases occur in children under the age of five [32]. If certain green juices could aid in cancer treatment, and/or minimize chemotherapy side-effects, and/or help with compliance problems [34], they could rise to true nutraceutical status.

Despite much hype given to anticancer greens [12], some controversy persists about juicing [13,35,36]. In the sole human trial of anticancer green juices we are aware of, subjects consumed 200 g of kale juice with a meal loaded with nitrates (400 mg), which lowered the postprandial blood levels of a carcinogen, N-nitroso-dimethylamine, in an open-label trial on subjects of unknown predisposition to cancer [37]. We performed an in vitro study in a Master’s thesis [37], showing that kale juice but not green leaf lettuce juice causes monocultured rat melanoma cells to stop proliferating, with no detriments from either juice on a nonmalignant cell type [11]. A mouse study also revealed an anti-genotoxic (anti-mutation) dimension to kale juice [38]. The juices of Brussel sprouts and red cabbage have likewise been reported [39,40] inhibitory on the chemo-induction of cancer in rats, and the juices of cauliflower and broccoli have been shown anti-proliferative in vitro on cancer cell lines HT29 [41] and MCF7 [42]. Then there are many studies of extracts (chemically defined fractions) [10,41], which are far different from what people drink. Some kale extracts have shown activity against *Caco-2* cells, which we use as a control cell line, yet the varied conditions between juices and extracts preclude close comparisons (per paragraph below) [10,43]. In addition, there are studies that argue against green juices being anticancer [13,35,36]. For instance, exposure of kale juice to gastric enzymes has ability to markedly degrade many of its phytochemicals [36]. Another study has shown that kale glucosinolate hydrolysates increase after digestive cocktail, but of uncertain consequence [44]. Furthermore, the juice of spinach has been reported [17] noxious to cultured human cells due to its high calcium-oxalate content.

Among the molecules highly enriched in kale (Scheme 1), the flavones/flavonoids are by-and-large too lipophilic to substantially transit the intestine in doses possible from drinking green juices [7,18]. This was shown first using monocultured human *Caco-2* cells with tight junctions on filter inserts, subsequently backed-up by ex vivo and in vivo paradigms [45,46]. Yet, a careful review of the literature showed us that one particular flavonoid, kaempferol, is of high enough concentration in *Brassica*, with stability against digestive enzymes, undergoing substantial unidirectional transepithelial transport to reach the basal compartment (blood side) of *Caco-2* monoculture models [45,46]. Kaempferol, to our knowledge, has not heretofore been singled out as possibly the only bioavailable anticancer flavonoid that makes kale juice a standout.

Several key methodological factors are important to emphasize about our model system. We knew from the outset that under certain conditions, *Caco-2* cells can behave as an alternative model of colon cancer [47]. Moreover, another report [48] co-culturing the same cell lines claims them to be normal enterocytes above normal cholinergic neurons, respectively. In developing our model system we therefore needed ways to express them as a normal enterocyte barrier adjacent to malignant neuroblastoma cells [48]. Namely: (1) our *Caco-2Bbe1* cells were grown 15 days to achieve static (actually non-metastatic) confluency, making them behave like normal enterocytes before treating them; (2) serum-depleted media was interjected apically to better model the absence of serum in the lumen; and (3) no differentiation agents were added to *SH-SY5Y*s, leaving them fully metastatic and therefore not converted to cholinergic neurons. Evidence that we had achieved the desired model is displayed by the two best known neuroblastoma sub phenotypes [49,50]: namely, catecholaminergic stellar *SH-SY5Y* monocytes [51] and the occasional *SH-SY5Y* neurospheres [52]. Neurospheres are loosely-attached spheroid bundles of cells, analogous in some ways to blastulas because the cells have stemness features [53]. Having found these sub phenotypes adds face validity of our model, as does the fact that the *Caco-2* enterocytes formed tight junctions [54,55]. This was all done in preamble to running the juice studies.

With model constraints in hand, we have determined the rank order of four green juices acting through a tight epithelial layer to inhibit the viability of neuroblastoma cells. Our hypothesis was that kale juice diluted to a degree expected in the intestinal lumen would be most potent in exerting transepithelial anti-cancer efficacy to inhibit the growth of the *SH-SY5Y* neuroblastoma cells. We expected the other juices would fall in line with a review of the anticancer literature for each juice: namely, dandelion [56,57] > green leaf lettuce [11] > spinach [1,27].

## 2. Materials and Methods

### 2.1. Cell Lines

**Scheme 2:**
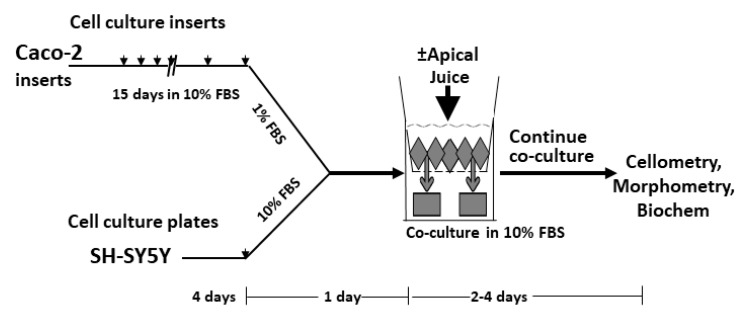
Co-culture model to study transepithelial effects of various juices. Essential components are apical side epithelial cells in communication with, by being overlaid on porous inserts about 1 mm from, a basolateral side of dividing neuroblastoma cells. (STEP-1) To set-up the apical side, human *Caco-2Bbe1* cells were plated on polycarbonate inserts (0.4-micron pores) in 24-well plates at 25,000/insert, with the intention to divide and differentiate in complete media to about day-11 when mature enterocytes normally differentiate, as depicted by the connected gray diamonds. The downward black arrows during the process (shown small and large) indicate media replenishment days before co-culturing (1 mL) as well as after combining the apical (0.5 mL) and basolateral (0.5 mL) compartments. The downward gray arrows demark the flow of juices. Until day-16, media replenishments were always made with complete media in 10% FBS. However, on day-16 lasting 24 h, the apical side was placed in 1% FBS media to better mimic the state of intestinal lumen (but not using FBS-free media, which we found caused *Caco-2Bbe1* detachment). Not shown are days for measuring transepithelial electrical resistance (TEER), which began daily from day-11. TEER readings started 20 min after moving the cells from the incubator. To ensure the model was mature required resistance >180 ohms/cm^2^, indicative of the formation of tight junctions to begin treatments. (STEP-2) On overall day-11, human *SH-SY5Y* cells were plated in separate 24-well plates in open wells at 50,000 per well with complete media (10% FBS), with expectation 4 days later of many more stellate monocytes and occasional neurospheres. The diagram portrays *SH-SY5Y* cells as gray squares after the inserts were transplanted, but for days prior, the *SH-SY5Y*s had already attained stellate shapes and rapid exponential cell divisions, which continued. (STEP-3) The co-cultures began on overall day-16. To start the co-cultures, the *Caco-2Bbe1* inserts in 1% FBS were transplanted atop the growing *SH-SY5Y* plates in complete 10% FBS media fed the same day. (STEP-4) One day after the co-cultures were established, on day-17, juice treatments began. The apical media was discarded without washing and replaced with complete media (0.5 mL in 10% FBS) prediluted with either a juice or PBS in the range 0.7–4.2% (vol juice/vol media). Only the apical side was treated; the basolateral compartment received neither media replenishment nor any direct juice treatment on this day or going forward. Immediately after the juices were added apically, baseline TEER readings started some 20 min after the plates came out of the incubator. After that, the co-cultures went back into the 5% CO_2_/37 °C incubator with no further daily treatments or media feedings. They were taken from the incubator to a sterile hood only for brief TEER readings and microscopy, and then some days later came the final measurements. Notice the time scale in the diagram changes in blocks of days 0–11, 11–15, 16, and 17–21. For more information, see a report by Louzao et al. [48], but we have made numerous changes to it [11,54,55].

The human neuroblastoma cell line, *SH-SY5Y*, and the human colon adenocarcinoma cell line, *Caco-2Bbe1*, were purchased from ATCC (Manassas, VA, USA). The *Caco-2Bbe1* subclone of *Caco-2* was selected because it is more uniform cell shape and lower mucus production than *Caco-2*. Both cell lines were maintained in identical media containing 10% fetal bovine serum (FBS) as previously described [55]. The growth curves of each cell line were regularly assessed to ensure the cells remained unchanged whether in monoculture or co-culture. A pictorial description of the co-culture process is shown in Scheme 2. It started from T75 flasks maintained in a 37 °C/5% CO_2_ incubator, passaged every 6 days in 15 mL of fresh media; roughly equivalent to passaging every time the cells reached 2.0 × 10^6^ cells/T75 flask or about 80% of maximal density. Passage numbers for experiments varied from 10–40. The *SH-SY5Y*s were replated in T75s at 1:4 dilutions and *Caco-2Bbe1* at 1:5 dilutions.

### 2.2. Juice Preparations

Two bundles of the leafy parts of vegetables were purchased at a local grocery store (Kroger, Clinton, MS, USA): (1) green curly kale, *Brassica oleracea* L. var. *sabellica*, product code #94627 from Braga Fresh Family Farms in Soledad, CA; (2) dandelion leaves, *Taraxacum officinale* Weber Ex F. H. Wig., product code #94615 from Lady Moon Farms in Florida; (3) spinach leaves, *Spinacia oleracea* L., product code #94090 from Cal-Organic Farms in Bakersfield, CA; and (4) green leaf lettuce, *Lactuca sativa*, product code #94076 from Earthbound Farm, San Juan Bautista, CA, USA. All had been freshly delivered to the store and were USDA-certified, non-GMO, and organic raised. In the laboratory they were washed thoroughly in a large, sterilized pan filled with deionized water and diluted soap (Dawn Ultra antibacterial hand soap, orange scent, Proctor and Gamble, Cincinnati, OH, USA). After rinsing away the soap with copious volumes of deionized sterile water in 7 cycles, the leaves were shaken and patted to dry with sterile towels and the final greens were adjusted to 438 g each. These were processed as previously described [11] using a Breville^®^ IKON BJE510XL (Sydney, Australia) centrifugal juicer. This brand juicer was common to many local stores and the only thing done differently from instructions was UV-sterilizing its components beforehand. The juice volumes ranged between 230–255 mL, with lettuce lowest (230 mL) and spinach highest (255 mL). These were mixed, aliquoted (40 mL) and frozen at −20 °C. No more than a month later, one tube of each vegetable juice was thawed and vacuum-passed through a 0.1-micron filter (Steri-Cup, Millipore, Burlington, MA, USA). Unfortunately, less than 30% of the volumes of each made it through the filters because they clogged. We used what came through, the assessments revealing small differences in filtrate volumes and pH: kale (7.0 mL, pH 5.0), dandelion (6.4 mL, pH 5.0), spinach (7.5 mL, pH 6.0), and lettuce (10.5 mL, pH 6.1). Each filtrate was then adjusted to pH 7.4 by dropwise addition (about 50 μL) of 0.6 M NaOH. Each juice was also measured by fluorescence at λ_ex_ = 260 nm, λ_em_ = 450 nm to ensure they were all roughly equivalent. They were also checked for sterility after 1:15 dilution by overnight incubation in complete DME/F12 media on MRS agar plates (no bacteria detected: see below). They were aliquoted and frozen at −20 °C before the cell biology studies began using multiple dilution doses.

As described in the Discussion, we performed calculations based on imaging the volume of the human GI tract [58]. These calculations indicate that the lowest (0.7%) and highest (4.2%) doses in our study equated to levels achievable in the human small intestine after a normal meal, as follows: low dose = 7 mL of juice containing 8.2 g total wet weight of each green; high dose = 42 mL of juice containing 49 g wet weight of each green. These doses are low compared to 200 g of kale (wet weight) in the juice consumed in a human clinical trial [37].

### 2.3. Co-Culture Plating and Treatments

Detailed methods for the co-cultures are in Scheme 2, but briefly, the *Caco-2Bbe1* cells were seeded at 25,000 cells/well on top of 0.4-micron inserts. After 6 days in the 37 °C/5% CO_2_ incubator, media replenishments began and continued every other day. None of the cells were observed to transit the inserts. Transepithelial electrical resistance (TEER) readings began on day-11. Based on TEER being >180 ohms/cm^2^, indicative of the formation of tight junctions, it was time to seed the *SH-SY5Y* cells. They were seeded at 50,000 *SH-SY5Y*s/well in open wells with 0.5 mL complete media in separate 24-well plates designed to eventually fit the inserts (Scheme 2). These grew 4 days before co-cultured with day-15 *Caco-2Bbe1* inserts. The inserts were transplanted above the *SH-SY5Y*s and the apical side subjected to 24 h in 1% FBS, a depletion designed to rid the lumen model of most FBS (to better match in vivo lumen). After this, the apical side received phosphate-buffered saline (PBS) or dilute juices (0.7–4.2%: *v*/*v*) made in re-completed media (10% FBS again). Once back in the 37 °C/5% CO_2_ incubator, the co-cultures were unperturbed except for measuring TEER daily until the endpoints. Parallel open-well monocultures were examined and photographed so that cell shapes and/or neurospheres were assessed, not doable non-invasively when the inserts were in place (Supplementary Figures S4 and S5).

### 2.4. TEER and Other Maturity Measures of the Caco-2Bbe1 Inserts

A sign of *Caco-2Bbe1* maturity is tight junctions, assessed by TEER using a calibrated Millicell^®^ ERS-2 system (Fisher Scientific, Waltham, MA, USA). The raw TEER values were adjusted by blank subtraction and correction for surface area of the filters (0.6 cm^2^). Only values >180 Ω cm^2^ resistance qualified for an experiment [59]. When the green juice experiments were complete, then the filters were dried, fixed in methanol, and stained sequentially with HEMA 3 solution-1 (Fisher CAT #122-911B: Eosin Y) and HEMA 3 solution-2 (Fisher CAT #122-911C: Azure A and Methylene Blue). After clearing away the unbound stain with water, the filters were subjected to light microscopy. This gave a final measure of their health and maturity, showing an absence of gaps in the layers as regularly spaced nuclei-stained cells with normal cytoplasm as stained by Azure A and Methylene Blue staining.

### 2.5. Comparative Role of Lactobacillus Fermentum

As mentioned above, all juices screened free of bacteria when diluted 1:15 in DME/F12 complete media, grown overnight, and [60] plated on MRS agar (ATCC, Inc.). However, we also treated the cell lines with a *lactobacillus* found in juice fermentation, to determine if any aspect of our juice findings were mimicked by adding bacteria. The bacteria chosen for this study was *Lactobacillus fermentum* Beijerinck (LF: ATCC^®^ 9338^™^), known active in spinach juice fermentation [60]. We hypothesized that if a contaminant *lactobacillus* played any role in distinguishing one juice from the other (especially suspicious of spinach) then with added LF treatments we should see partly the same result. LF was prediluted 1:15 in MRS broth and grown for 18 h at 37 °C. We separately compared a range of LF colony forming units (CFUs) and turbidities (OD 600), so to plate 1.0 × 10^7^ CFUs per 0.5 mL well on each human cell line in DME/F12/10% FBS. The cells were kept antibiotic-free in apical inserts and control open wells. LF was added at the same point in Scheme 2 when the *Caco-2Bbe1*s had high enough TEER to receive juice. We added LF directly to *SH-SY5Y* (alone) or *Caco-2Bbe1* (alone) and observed that the bacteria did not overgrow the wells or act overtly against either cell line over two days.

### 2.6. Automated Cell Counts by Cellometry

Automated cellometry^TM^ was performed according to Nexelcom Inc. on attached cells through a process of washing (saved floaters for later; below), detaching (0.25% porcine trypsin-EDTA), pelleting (5 min at 500× *g*), resuspending in phosphate-buffered saline (PBS), diluting 1:1 with trypan blue dye solution (Fisher), transferring to a 20 μL chamber in a hemocytometer slide, and placing them into the slides of a cellometer (Nexelcom, Lawrence, MA, USA) [55]. The instrument subtracted cellular debris simultaneously for counting live cells (trypan blue excluding), numbers of dead cells (trypan blue throughout), percent viabilities, and mean cell diameter. For most data in this report, the values are “total” per well, meaning from recombining the predominant “attachee” phenotype collected cells just after trypsin-EDTA digestion, with the “floaters” in the media + the wash in combined pellets. In samples where the microscopy beforehand showed an abundance of floaters (be they singlets or neurospheres), we modified the procedure to allow for separately pelleting the attachee vs. floater cells.

### 2.7. Metabolism of 3-(4,5-Dimethylthiazol-2yl)2,5-Diphenyl Tetrazolium Bromide

Other wells of cells were stained with a metabolic dye, MTT (3-(4,5-dimethylthiazol-2yl)2,5-diphenyl tetrazolium bromide). Reduction of yellow MTT to a purple formazan by enzymes supposedly confined in mitochondria is widely considered to measure cell health, and therefore the number of cells in a dish [61]. However, the MTT method is also known for a signature effect when uncoupled by flavonoid phytochemicals [62,63,64]. To show this, our cells were washed with PBS (0.5 mL) to separately collect (for later) the floaters. The attachees were diluted with 0.5 mL of PBS per well containing 50 μL of 5 mg/mL of MTT. These were wrapped in a dark 37 °C/5% CO_2_ incubator for 3 h. The reactions were stopped by solubilizing in solvent (4 mM HCl, 0.1% Nondet P-40; in isopropanol) using gentle agitation to mix. Plate readings (BioTek) of the reduced MTT dye were made at absorption peak λ590 nm minus reference at λ620 nm [65]. The results used blank media corrections.

### 2.8. Reactive Oxygen Species Assay

Reactive oxygen species (ROS) were measured in treated *SH-SY5Y* cells by the H2-DCFDA dye described earlier [66]. Briefly, *SH-SY5Y* cells were seeded in 96-well plates and 24 h later the cells were treated with low (0.7%) or high (4.2%) concentrations of kale, dandelion, lettuce, or spinach juice for another 24 h. Following juice treatments, the cells were washed with PBS and stained with 10 μM dichlorodihydrofluorescein diacetate (DCFDA) diluted in PBS and incubated at 37 °C for 30 min in the dark. After incubation, cells were washed three times with PBS. The DCF fluorescence was measured at excitation and emission wavelengths of 485 and 535 nm, respectively.

### 2.9. Cell Cycle Analysis

*SH-SY5Y* cells, seeded at 5.0 × 10^5^ per well in 6-well plates (3 mL), after 3 days in culture, were treated with kale, dandelion, spinach or lettuce juices at the same low or high dilutions mentioned above. Twenty-four hours later, the cell cycle distribution of the cells collected in each well (floaters and attachees together) was determined as described previously [11] (Abcam, Inc. reagents, Cambridge, UK). Post-treatment cells were harvested, fixed, and stained with propidium iodide solution containing RNAase A. This was followed by flow cytometric analysis (BD Accuri C6 Flow Cytometer). At least 5000 cells were assessed per sample in terms of propidium iodide fluorescence run at a slow flow rate (14 μL/min). Standard beads were used to calibrate the cell sizes (BD Biosciences).

### 2.10. Spectroscopic Measurement of the Juices

Juice’s steady state absorption spectra were monitored with a Cary 60 dual beam spectrophotometer (Agilent Technologies). Then, steady state fluorescence measurements were carried out with a Fluoromax-4 (Horiba Jobin Yvon, Piscataway, NJ, USA) spectrofluorometer. Diluted aqueous solutions (10 μL stock juice/990 μL water) of the juices were also taken through electronic spectroscopic measurements. FTIR spectra were collected from a Nicolet iS50 FT-IR Spectrometer which had the Advanced Thermo Electron Harrick ConcentratIR2 with diamond ATR crystal and liquid N2 cooled MCT (mercury-cadmium-telluride)-A Detector with CdTe window. The software for FTIR data collection was Omnic version 7.3 (Thermo Electron Corporation, Waltham, MA, USA) and the water spectra was subtracted from the samples for analysis using the software. Before the analysis, the instrument was purged with nitrogen for 15 min. As a reference, the background spectrum of air was collected before acquisition of the sample spectrum. To record a spectrum, 200 μL of sample was pipetted on the ATR crystal. Spectra recordings were at resolution of 2 cm^−1^, and 1064 scans were averaged for each spectrum (scan 4000–650 cm^−1^).

### 2.11. Chromatographic Measurement of the Juices

Previous reports [67,68] using high performance liquid chromatography (HPLC) have coupled electrospray ionization multi-stage mass spectroscopy (ESI-MS(n)) to reveal all the main flavonoids of kale, including 71 different glycosides. We sought to know which of these compounds best distinguishes kale juice from the other leafy vegetables and therefore passed each of our juices through a near identical HPLC column except without the ESI-MS(n) [67,68], since we only had an inline MD-4010-PDA absorption and FP-4025 fluorescence detector. The time difference between our two inline detectors was around 0.1 min. Specifically, we used a Jasco LC-NetII/ADC system with AS-4050 autosampler, PU4180 pump, CO-4061 column oven, and C-18 silica LC column (Jupiter 5 μm C18 300 Å, Column 250 × 4.60 mm i.d.; Phenomenex, Torrance, CA, USA). Diluted aqueous solutions of each juice (10 μL stock/990 μL water) were injected (20 μL) at a flow rate of 0.5 mL/min in methanol/water (50:50) as mobile phase. We detected many of the peaks seen earlier with ESI-MS(n) and used purified kaempferol as a positive internal standard flavonoid, and daidzein as a negative standard flavonoid (not known to exist in our juices) (Sigma Aldrich, Inc., St. Louis, MO, USA). Three or more chromatographs of each sample were acquired to assign peak retention times in line with the chemical identities [67,68].

## 3. Results

**Figure 1:**
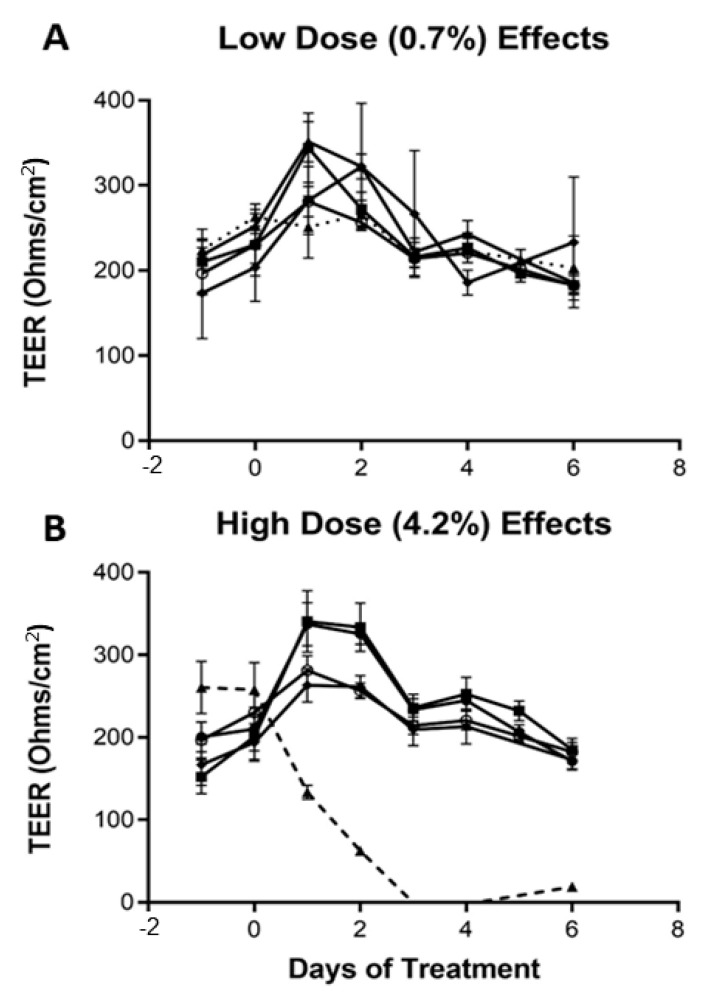
Effects of apical vegetable juices on trans-epithelial electrical resistance (TEER) of *Caco-2Bbe1* inserts above *SH-SY5Y* cells. Shown are seven consecutive daily TEER readings on the inserts of co-cultures fed on day-0 with the shown dilutions of either phosphate-buffered saline (PBS open circles, solid line), kale juice (closed squares, dashed line), dandelion juice (closed triangles, dashed line), spinach juice (open triangle, dotted line), or lettuce juice (closed diamonds, solid line). The readings began 15 days after seeding the *Caco-2Bbe1* on inserts, minutes after the apical media had been switched to 1% FBS and the inserts had been transplanted above growing *SH-SY5Y*s in 0.5 mL complete media basolateral (Scheme 2). Twenty-four hours hence, day-0, the apical media was replenished to complete 10% FBS media plus either phosphate-buffered saline or the juices to final dilutions (*v*/*v*) of 0.7% (Panel **A**) or 4.2% (Panel **B**), not changing the basolateral media at any point, and TEER readings continued. Values represent the mean ± SEM (*n* = 4–6 experiments per condition). Asterisks indicate statistical significance by two-way ANOVA, multiple comparisons: kale-high vs. saline (*p* = 0.065 at 1 day, *p* = 0.019 at 2 days), dandelion-high vs. saline (*p* = 0.048 at 1 day, *p* = 0.008 at 2 days).

The initial investigations confirmed that *Caco-2Bbe1* inserts developed tight junctions before additions and remained at high TEER after juice additions. As shown in Scheme 2, the “normal pattern” started with rising TEER from day-16 followed by a gradual return to baseline, always above an established cut-off of 180 Ω cm^2^ for tight junctions lasting to day-22 after juices [59] (Figure 1). However, two conditions varied from this normality. The first was 4.2% spinach juice compared to 4.2% saline, which caused TEER to drop steadily to zero by day-19 (Figure 1B). This fall in TEER by spinach juice corresponded with large gaps appearing between the *Caco-2Bbe1* cells. There was also evidence of spinach juice toxicity seeping through to the *SH-SY5Y* cells (described later). We were unable to culture bacteria from the 4.2% spinach wells and thus the phenomenon was not due to contaminant microbials. Moreover, wells treated with spinach-low (0.7% juice) appeared normal out to 22 days. Another factor was that 4.2% spinach was not mimicked by added lactobacillus (described below). Most poignantly, there appeared to be oxalate crystals in the 4.2% spinach wells but not the 0.7% spinach wells. Meanwhile, the other change from normal was a positive effect with 4.2% kale and 4.2% dandelion juices after 1- and 2-days treatment (Figure 1B). This TEER rise was statistically significant compared to no juice by two-way ANOVA with multiple comparisons (kale-high *p* = 0.065 at 1 day and *p* = 0.019 at 2 days, dandelion-high *p* = 0.048 at 1 day and *p* = 0.008 at 2 days). Aside from these exceptions, the other juice additions followed the normal pattern, i.e., no effects for kale-low (0.7%), dandelion-low (0.7%), lettuce-high (4%), lettuce-low (0.7%), and/or spinach-low (0.7%) juices (Figure 1A,B).

**Figure 2:**
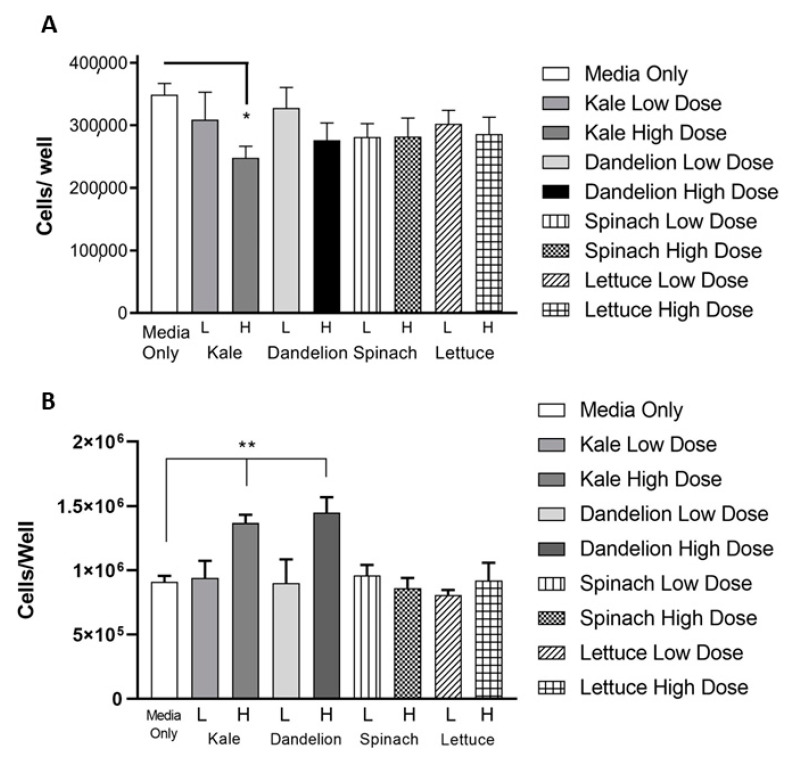
Effects of apical vegetable juices on cell counts by cellometry as per Scheme 2 and Figure 1, harvested after 3 days in co-cultures. The upper panel (**A**) shows *SH-SY5Y* total cell numbers taken by pooling attached and floaters from the co-culture well bottoms. The lower panel (**B**) shows epithelial carcinoma, *Caco-2Bbe1*, total cell numbers taken by pooling attached and floaters from the co-culture inserts. The graph shows total cells whether trypan blue excluded or not, however the average percent viabilities values (trypan blue excluding) were as follows: from phosphate-buffered saline-high apically, 74.4% viability; *SH-SY5Y* from kale-low apically, 79.4% viability; *SH-SY5Y* from kale-high apically, 81.4% viability; *SH-SY5Y* from dandelion-low apically, 82.4% viability; *SH-SY5Y* from dandelion-high apically, 79.3% viability; *SH-SY5Y* from spinach-low apically, 63.0% viability; *SH-SY5Y* from spinach-high apically, 69.7% viability; *SH-SY5Y* from lettuce-low apically, 78.3% viability; *SH-SY5Y* from lettuce-high apically, 69.6% viability; *Caco-2Bbe1* from phosphate-buffered saline-high apically, 72.7% viability; *Caco-2Bbe1* from kale-low apically, 73.2% viability; *Caco-2Bbe1* from kale-high apically, 75.1% viability; *Caco-2Bbe1* from dandelion-low apically, 74.0% viability; *Caco-2Bbe1* from dandelion-high apically, 74.6% viability; *Caco-2Bbe1* from spinach-low apically, 72.5% viability; *Caco-2Bbe1* from spinach-high apically, 50.8% viability*; *Caco-2Bbe1* from lettuce-low apically, 71.3% viability; *Caco-2Bbe1* from lettuce-high apically, 74.5% viability. Asterisks mark the statistical group differences from appropriate phosphate-buffered saline controls. Graph bars represent the mean ± SEM (*n* = 4–5 experiments per condition). Details comparing the low (0.7%) and high (4.2%) doses to what human’s drink are discussed in the methods section. * *p* < 0.05, ** *p* < 0.01, compared to media-only by two-tailed analysis.

Next, the main goal of determining if the juices can act transepithelially on underlying neuroblastoma *SH-SY5Y* cells in the co-culture model. The results of Figure 2 indicated that three days after the juices were applied, the high dose (4.2%) of kale juice was uniquely potent. Dandelion-high juice (4.2%) was a close second, but statistically none of the other juices, even spinach high dose (4%), caused a significant decline in *SH-SY5Y* numbers or percent viability three days after addition (Figure 2A). Only the high dose (4.2%) of kale juice did this. At the same time, the finding of fewer *SH-SY5Y* cells after three days of transepithelial high dose kale juice was not accompanied by low percent viabilities in the remaining cells, which remained good at 81.4% viability (data in legend of Figure 2A). This meant that kale juice acted cytostatically rather than via overt cytotoxicity. The evidence for mainly a cytostatic mechanism was extended at five days in co-cultures treated with kale juice (Supplementary Figure S2). However, by that time, kale was no longer so unique in crossing the epithelium. Namely, after five days co-culture, spinach’s high dose toxicity (Figure 1) seeped through to cause *SH-SY5Y* cell numbers to decline. After five days, the dandelion-high dose (4.2%) had also amplified to become equally anti-neuroblastoma as kale-high effect (Supplementary Figure S2).

Figure 2B shows that the high doses of kale and dandelion juices (4.2%) in the same co-cultures also acted pro-proliferatively on the inserts with *Caco-2Bbe1* cells. This was reminiscent of the higher TEER elicited uniquely from the kale and dandelion juices in Figure 1. In regard to *Caco-2Bbe1* percent viabilities, none of the juices modified with exception again that the spinach-high dose killed *Caco-2Bbe1* cells (percent viability at 50.8%; see data in legend of Figure 2). The complexity of this data begged the next question whether the pro-*Caco-2*/TEER effects were in any way causally linked to the anti-neuroblastoma effect seen with kale and dandelion juices on the basal-side (Figure 2A). Furthermore, it forced us to contemplate whether the model system was unidirectionally examining transepithelial effects, or whether the juices might alter crosstalk between two adjacent cancer cell lines?

**Figure 3:**
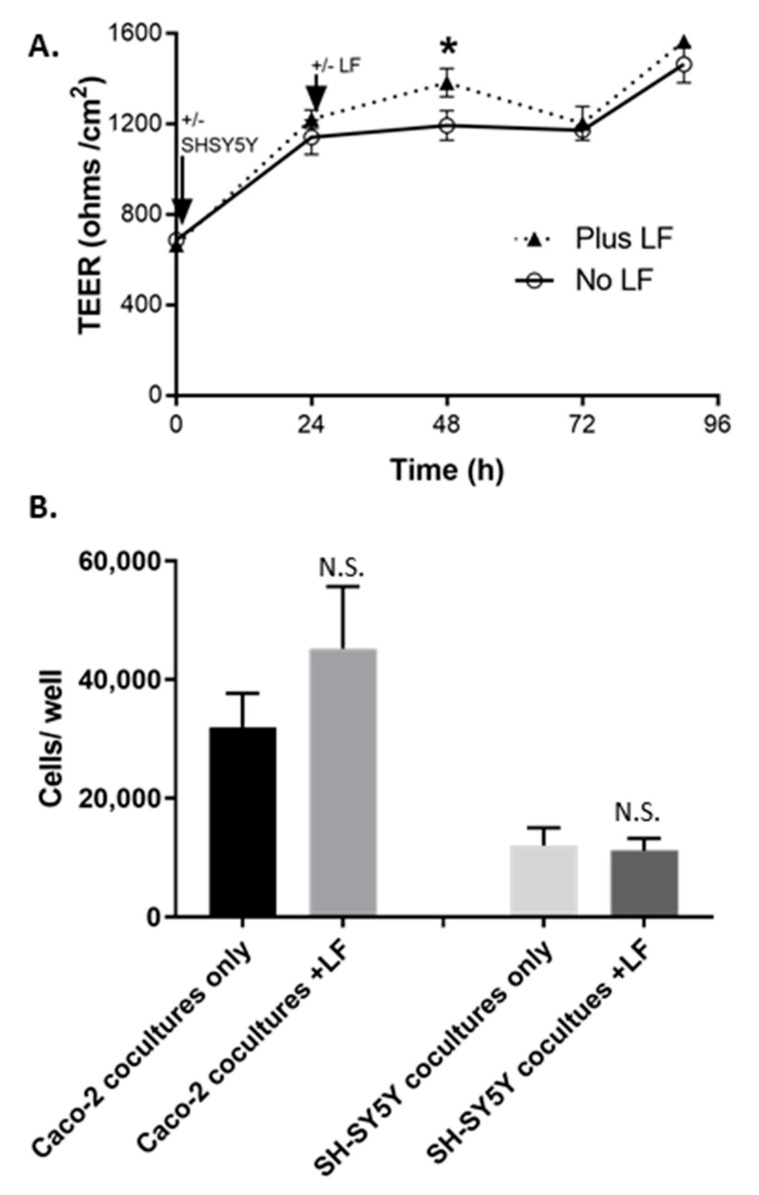
Effects of the apical addition of *Lactobacillus fermentum* (LF) on the co-culture model in terms of (**A**) TEER and (**B**) cellometry of the two cell lines. Setting-up the model followed Scheme 2, except in place of juice was added 1.0 × 10^7^ colony-forming units of the lactobacillus or complete media without the bacillus per well. A three-day series of daily TEER measurements ensued as in Figure 1. At the end of three days, the cells were harvested from the inserts (*Caco-2Bbe1*s) and from underneath in the wells (*SH-SY5Y*s) and counted by cellometry as in Figure 2. Points represent mean values ± SEM (*n* = 4–5 experiments per condition). The asterisk indicates *p* < 0.05 by two-tailed analysis.

To address this and untangle if raising TEER necessarily leads to *SH-SY5Y* cytostasis, we turned to a completely different treatment modality (Figure 3) that we knew from previous studies would cause pro-TEER effects, namely adding *Lactobacillus fermentum* (LF) [69]. By adding LF (alone) on the apical side of the co-cultures, and causing, just as kale and dandelion juice had done, a rise in *Caco-2Bbe1*′s growth and TEER—but without consequence on *SH-SY5Y* cells—we hypothesized to dissociate *Caco-2Bbe1*/TEER from *SH-SY5Y* cytostasis. Such would address the concern that merely by raising TEER, rather than direct phytochemical action, had acted on the *SH-SY5Y* cells (Figure 2). The data met our hope (Figure 3). That is, LF raised TEER (as expected and similar to Figure 1B of kale and dandelion) and also raised *Caco-2Bbe1* numbers (similar to Figure 2B of kale and dandelion). But, LF had no effect on underlying *SH-SY5Y* cells (dissimilar to Figure 2A of kale and Figure S2 of dandelion). Therefore, LF showed no anti-neuroblastoma activity either directly or in co-cultures (Figure 3B). It only raised TEER slightly (Figure 3A), in keeping with an earlier report [69]. Raising *Caco-2Bbe1* TEER therefore does not necessarily cause *SH-SY5Y* cytostasis. Why the kale and dandelion juices, or LF for that matter, raise *Caco-2Bbe1*/TEER, is a topic for future study. But, at least this moved the experiments forward with a specific focus on kale juice.

**Figure 4:**
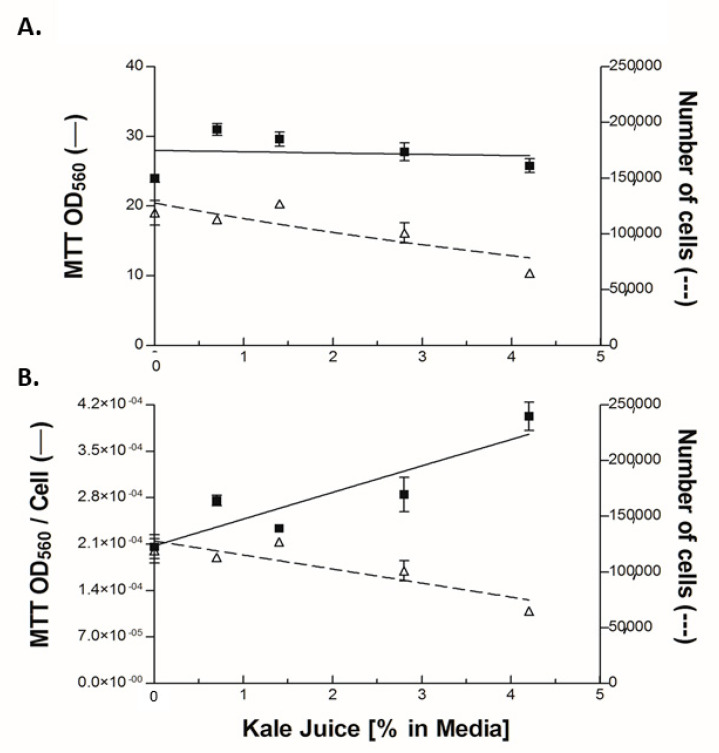
Dose response effects of apical kale juice on *SH-SY5Y* cells in the co-culture model (following Scheme 2 and Figure 1) after 3 days comparing numbers of cells by cellometry vs. reduction of MTT metabolic dye marker. All wells had equal numbers of cells when making kale juice dilutions (or adding equal volume vehicle). Three days later, panels (**A**) and (**B**) contrast the results expressed in panel A as MTT per well (left Y-axis) or expressed in panel B as MTT per cells in the well (left Y-axis). Points represent mean values ± SEM (*n* = 4–5 experiments per condition) and the lines were drawn by linear regression in GraphPad Prizm.

To more fully understand kale juice’s activity on *SH-SY5Y* cells in co-culture, two different cell number measurements were used: cellometry as above (Figure 2 and Figure 3) plus a “metabolic way” using MTT formazan dye [65]. The results shown in Figure 4A revealed a straight dose decline with kale in terms of cells/well, fully in agreement with Figure 2. Again, the lowered *SH-SY5Y* cell numbers were unchanged in percent viable cells (always >80% viability). By contrast, when examining metabolism, there was no decline in MTT per well (Figure 4A). In fact, converting the same data to MTT/cell showed that kale juice dose-responsively increases the MTT parameter (Figure 4B). This was not the only time this paradox loomed (see the slow exponential decline curves in monocultured *SH-SY5Y* cells in Supplementary Figure S1). The literature widely claims that MTT reduction takes place via mitochondrial reductase enzymes. Hence, to convert MTT to a purple color theoretically would require more mitochondria—which by adding kale juice seems highly unlikely. A subset of the literature shows, however, that purified flavonoids lead to an “artifactual” reduction of MTT dye [61,62,63,64]. To observe a full comparison of this experiment in the *Caco-2Bbe1* cells, please see Supplementary Figure S1 (less dichotomous).

**Figure 5:**
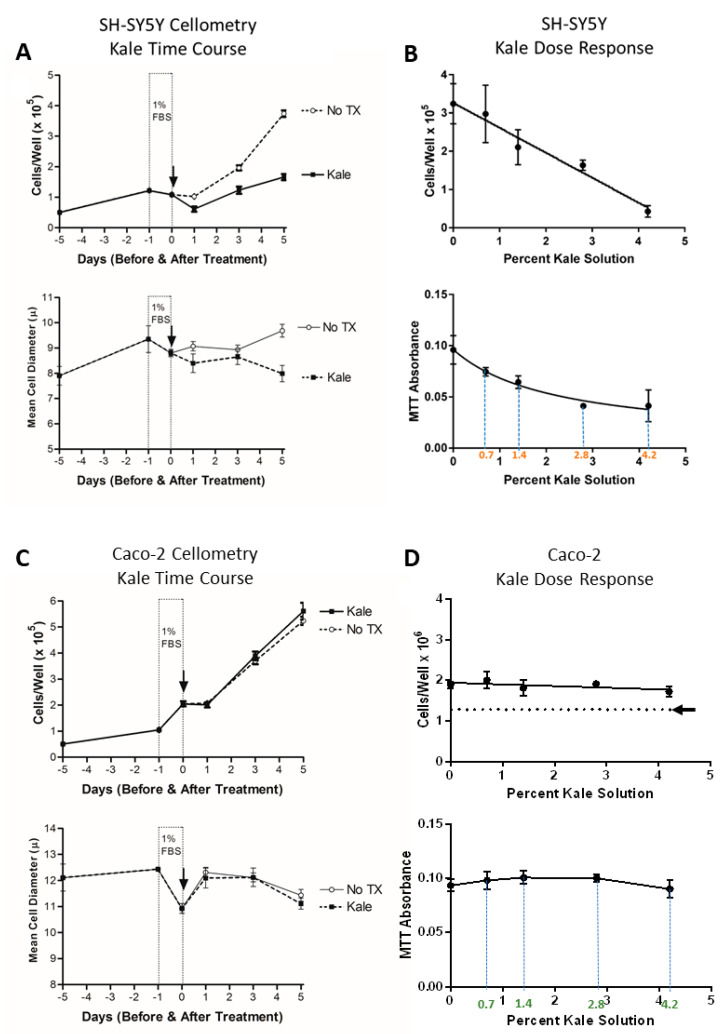
Kale juice time course and dose response curves for monocultures of *SH-SY5Y* (**A**,**B**) and *Caco-2Bbe1* (**C**,**D**), comparing total cellometry counts, mean cell diameters, and MTT metabolism values. The same analytical methods were used as in Figure 4 except now in monocultures. After plating and growing the cells in 24-well plates for four days, they were switched to 1% FBS media for 24 h, and then given complete media replacements the next day with up to either 4.2% kale juice or 4.2% PBS and monitored over five more days: A-upper panel, *SH-SY5Y* cell counts; A-lower panel, *SH-SY5Y* mean cell diameters; C-upper panel, *Caco-2Bbe1* cell counts, C-lower panel, *Caco-2Bbe1* mean cell diameters. B-upper panel, *SH-SY5Y* cell counts; B-lower panel, *SH-SY5Y* MTT metabolism; D-upper panel, *Caco-2Bbe1* cell counts, D-lower panel, *Caco-2Bbe1* MTT metabolism. Arrows are shown to point the cell numbers present at day-0 of treatment (determined in adjacent wells). Points represent mean ± SEM (*n* = 4 experiments per condition) and the curves were drawn in GraphPad Prizm.

It was next of interest to examine monocultures in terms of dose responses, times, and mechanisms (i.e., cell cycle and ROS). Shown in Figure 5A,B is results using monocultures of *SH-SY5Y*s given kale juice, and Figure 5C,D show monocultures of *Caco-2Bbe1*s given kale juice. Both *SH-SY5Y* cell numbers and mean diameters diminished over time with 4.2% kale juice, suggestive of not only cytostasis but also neuroblastoma cell shriveling (Figure 5A lower panel). The control *SH-SY5Y*s without kale treatment, however, grew rapidly after one day in 1% FBS, maintaining good diameters (Figure 5A,B). As in Figure 4, a linear dose-dependent drop emerged from kale juice in open well *SH-SY5Y*s after 2 days (Figure 5B upper panel). By contrast, a logarithmic dose response decline was observed in response to the same kale doses in *SH-SY5Y* MTT/well (Figure 5B lower panel, and Supplement Figure S1). Overall, the pattern of monoculture *SH-SY5Y*s was therefore to halt cell divisions in response to one day in 1% FBS (Figure 5A) and then resume after the 10% FBS was restored; but given the presence of 4.2% kale juice, even the return of 10% FBS was negated by kale juice. Comparing the magnitude here due to 4.2% kale after 2 days in Figure 5B (87% drop in cell numbers and 50% drop in MTT by day-5) vs. the magnitude due to 4.2% apically in the co-culture model in Figure 3 (45% drop in cell numbers and 12% drop in MTT), we see that the dose response curve is shifted rightward about 50% when the layer of *Caco-2Bbe1* inserts is transplanted. This suggests that about 50% of the anti-neuroblastoma activity of kale passed through the *Caco-2Bbe1*s.

Figure 5C,D concern *Caco-2Bbe1* responses in monocultures exposed to kale juice. Keep in mind that these monocultures were neither receiving cross talk from *SH-SY5Y*s nor had achieved tight junctions or a plateau phase of cell growth. Thus, the cell conditions are highly different here than in Figure 1. The main effect came from 24 h in 1% FBS, rather than from kale juice (Figure 5C lower panel). That is, the growth rate of untreated *Caco-2Bbe1*s continued unabated during 1% FBS but there was a drop in mean cell diameter with or without added kale juice (Figure 5C lower panel). Afterwards, the rate of *Caco-2Bbe1* numbers sped up whether juice was applied or not, in line with 10% FBS being restored. Whether by cell counts or mean cell diameters, the growth rates of these *Caco-2Bbe1* were identical in kale juice (4.2%) or vehicle (4.2% PBS). Besides this, the dose response curves were flat (non-effective) for kale juice in *Caco-2Bbe1* cells after 2 days, also after 5 days (Supplementary Figure S1 in terms of either cells/well or MTT metabolism/well). Thus, the reader is reminded that in growth phase *Caco-2Bbe1*s act as adenocarcinoma cells, whereas on the inserts in Scheme 2 they represent normal gut epithelial cells. As such, there seems no effect of kale juice on the adenoncarcinoma state of *Caco-2s*.

**Figure 6:**
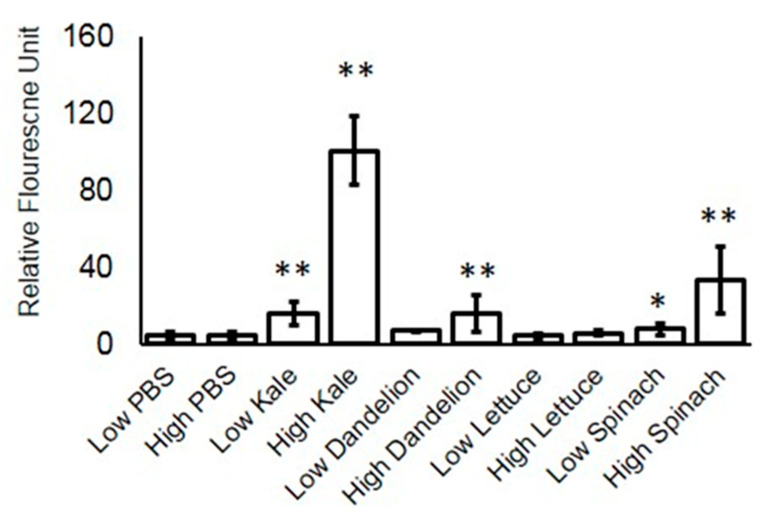
Measurement of reactive oxygen species (ROS) in *SH-SY5Y* cells after treating with kale, dandelion, spinach, and lettuce juices. Cells were seeded in 96-well plates and treated with the various dilutions of vegetable juices for 24 h. After treatment, the cells were washed with PBS and stained with 10 μM dichloro-dihydro-fluorescein diacetate (DCF-DA) diluted in PBS and incubated at 37 °C for 30 min in the dark. After incubation, the cells were washed, and cellular ROS production was measured by the amount of fluorescence (λ_ex_/λ_em_ = 485/535 nm) detected on a fluorescent plate reader. Data expressed as relative florescence unit (RFU). Data represents mean values of *n* = 8 ± SD. Statistical significance was determined by student’s t-test (* *p* ≤ 0.05, ** *p* ≤ 0.01).

It has been reported with purified phytochemicals [31] that ROS plays a role in mediating anti-cancer effects by manipulating different molecular pathways such as cell cycle arrest, growth inhibition, and downregulation of cancer stemness. To investigate if ROS was involved in the cell growth inhibition of the juices, intracellular ROS was therefore determined by using fluorescence dye DCF-DA after treating *SH-SY5Y* cells in monocultures. In Figure 6, the ROS production of *SH-SY5Y* cells is shown over 24 h in response to different juices in open wells. The results showed high dose kale (4.2%) with a robust rise in ROS compared to all other conditions. Smaller, statistically significant elevations in ROS occurred from low kale, high dandelion, low spinach, and high spinach compared to low dose PBS. By its magnitude, the results implicate ROS in the anticancer effects mediated by kale juice in these neuroblastoma cells (Figure 6).

It has also been reported [31] that intracellular ROS accumulation triggers cell cycle arrest, thereby inhibiting the growth of highly proliferating cancer cells. We next examined if the juice treatments also impacted the cell cycle. Cell cycle analysis was performed in *SH-SY5Y* monocultures after 24 h of treatments with PBS or the vegetable juices. The cells were harvested and fixed in 66% ethanol, stained with propidium iodine, and analyzed by fluorescence flow cytometry. Each experiment at low (0.7%) and high (4.2%) juice was performed twice, and the values averaged. Spinach was again problematic. At the high dose of spinach, there was such an abundance of oxalate crystals that they precluded proper fluorescence analysis (again also showing high dose spinach toxicity). However, the other juice effects were clear. Table 1 shows percent cells in S-phase cycle, obtained by propidium iodine staining and flow cytometry of *SH-SY5Y* cells treated with different juices. Kale juice, alone, consistently increased this parameter compared to vehicle (and other) treatments. Low dose kale juice rose 18.0% in S-phase cells while high dose kale juice rose 71.9% in S-phase cells compared to vehicle controls. There was no change in cell numbers or overall uptake of propidium iodine. The results can be interpreted as S-phase arrest due uniquely to kale juice. It places cell cycle arrest in uniqueness with oxidative stress/ROS induction as key elements of the kale juice anticancer treatments.

### 3.1. Chemical Analysis of the Juices

#### 3.1.1. Steady State Absorption and Fluorescence 

**Figure 7:**
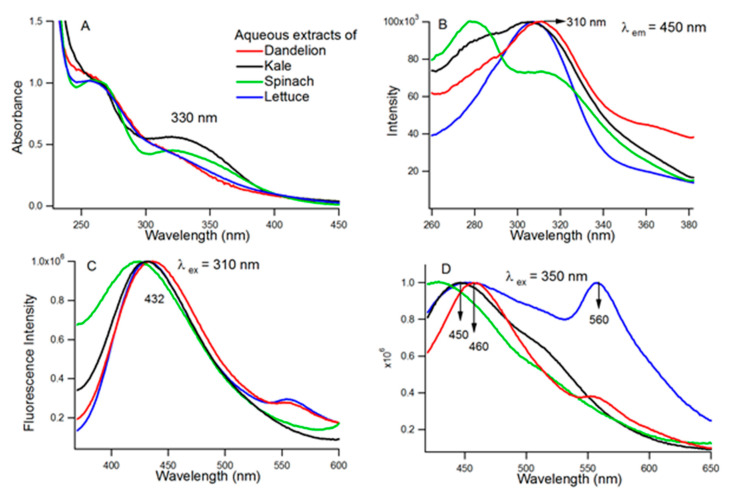
Normalized absorption (**A**) along with fluorescence excitation (**B**) and emission (**C**,**D**) spectra of the vegetable juices, λ_em_ = 450 nm (**B**), λ_ex_ = 310 nm (**C**), λ_em_ = 350 nm (**D**). The excitation and emission slit widths were 3/3 nm.

Figure 7A presents absorption spectra of the vegetable juices. Kale and spinach displayed an absorption band maximum at 330 nm, unlike lettuce and dandelion, which suggests some type of chromophores in kale and spinach juices that undergo a discrete *n* to π* transition compared to dandelion and lettuce aqueous extracts. The wavelength of maximum excitation ($\lambda_{\mathrm{ex}}^{\max}$) at λ_em_ = 350 nm, was found to be ~310 nm in (Figure 7B) for all juices except lettuce where the main excitation occurred at 280 nm. Figure 7C,D display the fluorescence emission spectra of the vegetable juices with excitation wavelengths λ_ex_ = 310 nm and 350 nm, respectively. The $\lambda_{\mathrm{em}}^{\max}$ (wavelength at emission maxima) of the main fluorophore in spinach was observed at 423 nm (when λ_ex_ = 310 nm, Figure 7C), which is ~9 nm blue shifted from the other juices ($\lambda_{\mathrm{em}}^{\max}$ is ~432 nm). Moreover, lettuce and dandelion juices showed a shoulder emission peak at 560 nm, which is even more prominent in lettuce when λ_ex_ = 350 nm (having the main emission at ~450 nm for lettuce and kale and the main at ~460 nm for dandelion). It is pertinent to mention that absorption spectroscopy pertains to the ground state characteristics (Figure 7A) whereas fluorescence excitation spectra looks at the excited states of the fluorophores (Figure 7B) [70], which is responsible for the difference in $\lambda^{\mathrm{abs}}$ and $\lambda_{\mathrm{ex}}$ respectively.

#### 3.1.2. Vibrational Spectroscopy

**Figure 8:**
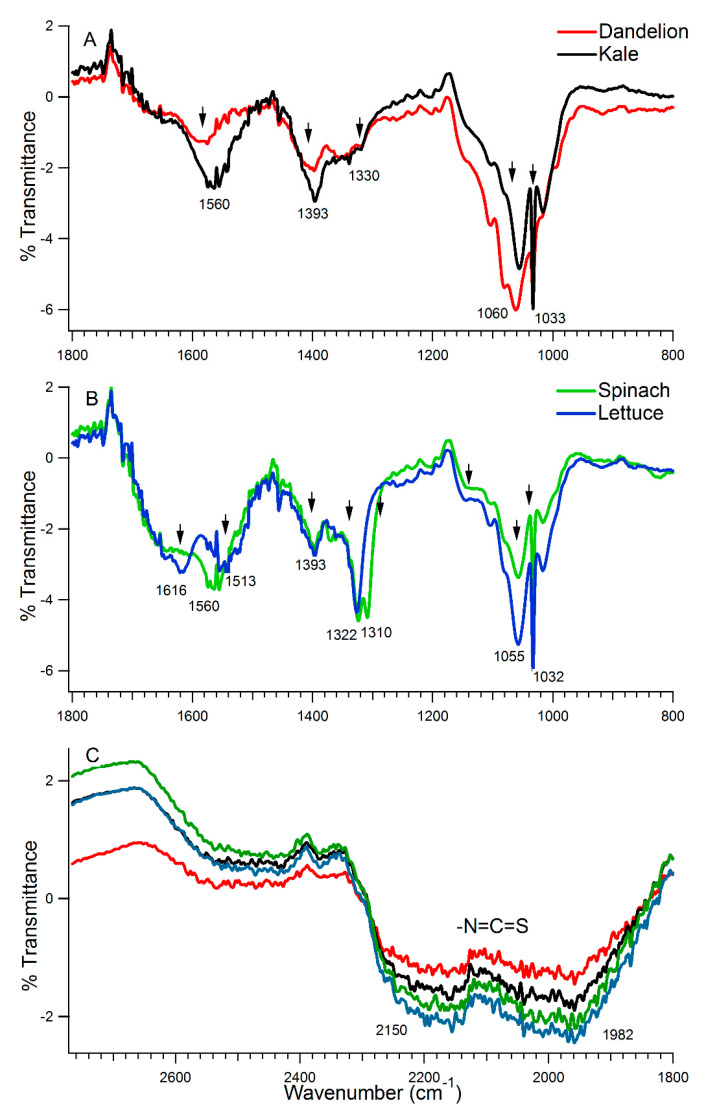
Attenuated Total Reflection–Fourier Transform Infra-Red (ATR-FTIR) spectra (water subtracted) of aqueous extracts of leaves of (**A**): kale (black), dandelion (red); (**B**): spinach (green) and lettuce (blue) in the region 800–1800 cm^−1^; and (**C**): The fingerprint area of isothiocyanates (in the region 1800–2800 cm^−1^) for all the extracts. The full scans of the raw extracts are provided in Figure S3.

Since the juices were in aqueous state, -OH vibrational bands (stretching and bending) existed in all FTIR spectra. By way of orientation, the characteristic water bands at ~3330 cm^−1^, related to stretching vibration of O-H groups; and at 1693 cm^−1^ due to O-H in-plane bending vibration and a coupling of the two at 2294 cm^−1^. When these water spectra were subtracted from those of the aqueous juice molecules, care was taken to not overlook ATR-spectrum containing other contributions. Supplementary Figure S3 shows the full FTIR scans while Figure 8 presents the FTIR spectra after water subtraction. Resemblances between kale and dandelion are evident (Figure 8A), and between lettuce and spinach (Figure 8B). Table 2 lists the more obvious IR band frequencies and the structures they connote by strength in each juice (bands taken from Figure 8). For instance, according to Kniseley, R. N. et al. [71], prominent doublets near 2150 cm^−1^ in the infrared spectra connote isothiocyanates (Scheme 1), which matched bands in all our juices in Figure 8C. Plus, the cumulative double bond system of -N=C=S in isothiocyanates would give rise to a bending vibration of the -CH bond (adjacent to the -NCS system) around 1318–1347 cm^−1^ [72]. That this was strongly observed in the spinach and lettuce juices (at ~1322 cm^−1^), but only a weak one for kale and dandelion (~1330 cm^−1^), suggests that categorically different isothiocyanates exist in spinach and lettuce compared to kale and dandelion juices.

The IR region between 1542 to 965 cm^−1^ is referred to as the “fingerprint” region [73,74] because it provides information about functional groups of organic compounds such as sugars, alcohols, and organic acids in the juices [73,74]. This includes various IR bands, including those corresponding to the vibrations of C–O, C–C, C–H and C–N bonds, which occur in this region (indicated in Table 2). Among the four juices, kale showed the strongest vibrational bands at the –OH in-plane bending (attributing likely to polyphenols) and S=O stretching (attributing likely to sulforaphane) (Scheme 1). Also of major importance is a telltale band at 1322 cm^−1^ which denotes the signature for oxalate—only seen in spinach juice (Figure S3) [27]. This fit the many green crystals seen earlier in the cell biology studies of only spinach juice treatments.

**Figure 9:**
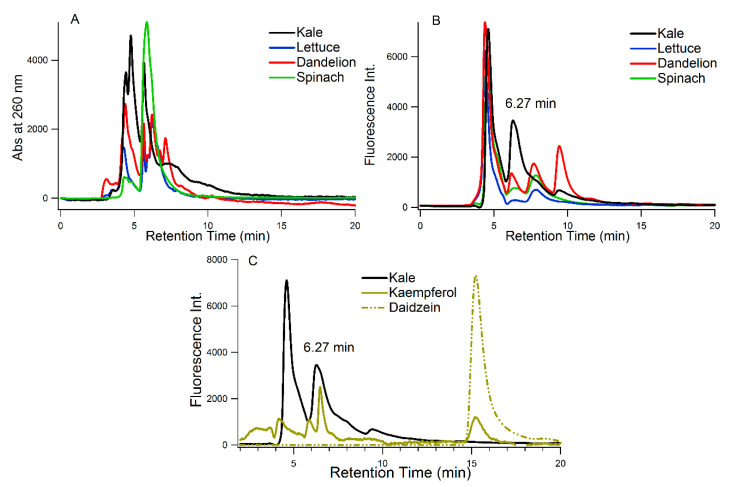
Typical chromatograms of the HPLC analysis of the juices of dandelion, lettuce, spinach, and kale using 50% methanol/H_2_O mobile phase on a C18 column with flow rate 0.5 mL/min. Panel (**A**) shows absorption (λ_abs_ = 260 nm) and panels (**B**) and (**C**) show fluorescence (λ_ex_/λ_em_ = 350/450 nm) measurements made using Jasco inline MD-4010-PDA and FP-4025 fluorescence detectors, respectively, where the time delay between the detectors was in the range of ~0.1 s.

HPLC analysis was next performed on the juices with a C18 silica column and two inline detectors. The mobile phase combined methanol and water (50%) to provide a dynamic range of dipolar and hydrogen bonding interactions which indicated as multiple peaks (Figure 9) [75]. Hydrophilic molecules eluted earlier than hydrophobic molecules due to reverse phase HPLC procedure. Usually, all the aromatic compounds absorb light at 260 nm, while a few are also fluorescent. As shown in Figure 9A, the vegetable juices showed several aromatic absorption peaks, differing in solubility but not much in overall concentration. Some peaks also exhibited fluorescence (Figure 9B) where all the juices exhibited a peak around 4.35–4.55 min. Kale uniquely showed a second relatively prominent fluorescent peak at 6.27 min. The main peak of standard kaempferol at 6.3 min overlapped well with the kale peak (Figure 9C). This finding agreed with the dichotomous observations in the earlier FTIR work (Figure 8B). By contrast, our more hydrophobic flavonoid standard, daidzein, eluted much later than any fluorescent molecules in our juices. We did not pursue other external standards to fully identify the main HPLC peaks at 6.2, 7.7, and 9.4 min because these were less prominent in kale juice and the abundance of the kaempferol peak in kale juice seemed to settle our main question about the uniqueness of kale.

## 4. Discussion

We have had to consider three main categories of potential anticancer phytochemicals in our studies, as shown in Scheme 1: isothiocyanates, flavonoids/flavones, and stilbenes [7,19,68,76]. Additional candidate molecules certainly exist (i.e., phenolic acids [20], indoles [77], carotenoids [78], and terpenoids [7]), but their anticancer contributions are less well documented. Starting with the glucosinolates (Scheme 1) [8,44,79], which are said to chemically distinguish *Brassica* from other vegetables [80], it is known that the metabolism of glucoraphanin leads to a major isothiocyante called sulforaphane (SFN) [3,4,8,81]. SFN is known to have anti-proliferative properties on brain cancer cells in vitro, by at least three signaling pathways: namely, induced nuclear factor erythroid 2-related factor 2 (Nrf2), activated extracellular signal-regulated kinase 1/2 (ERK1/2), and down-regulated matrix metallopeptidase 2 (MMP2) and Cluster of Differentiation 44 protein (CD44v6) [8]. SFN also leads to high reactive oxygen species (ROS), mitochondrial perturbations, and apoptosis in colon cancer cells in vitro [8]. SFN therefore must be considered in interpreting our good findings with kale juice, but it was also identified at high concentration in our green leaf lettuce juice (Table 2). SFN arises when glucoraphanin is enzymatically hydrolyzed (probably during juicing) to release the plant enzyme, myrosinase [3,4,8,81]. However, kale also has the flavonoids and stilbenes to consider (Scheme 1) [18,35,82]. Kale has at least 71 flavones/flavonoids/hydroxycinnamics inclusive of glycosides of quercetin, kaempferol, and isorhamnetin [67]. Most of them, except for kaempferol, have the problem of being difficult for uptake from the gut [67,68]. Resveratrol is the best known stilbene, very enriched in grapes [6,83]. It is known to elicit cell cycle arrest and apoptosis against cancer cells in vitro [29]. In kale, both resveratrol and its dimethyl analog, pterostilbene, are thought to coexist [84]. Because pterostilbene causes cell cycle arrest in cancer cells through reducing cyclin A and cyclin E [16,85,86], it too could play a role in the anticancer properties of kale juice in our experiments.

To our knowledge no one has previously co-cultured epithelial cells on inserts above cancer cells to directly determine the transepithelial activity of any anticancer agents, or juices, on any tumor cells. Smaller steps have been taken as described in the Introduction, and we must acknowledge the inspiration drawn from a previous model—in a very different setting—described by Louzao et al. [48]. Louzao’s group cocultured the same cell lines but to study the diarrhetic role of normal enteric neurons [48]. We have considerably modified that design [54] to create a novel system focused on anticancer agents. Among many changes, our *Caco-2Bbe1* cells were cultured long enough to transform into small intestine-like enterocytes rather than remaining as dividing adenocarcinoma cells [87]. We further ensured they attained high enough TEER so their tight junctions remained intact throughout the treatments [44,45,59,88]. Of course, other co-culture systems can be imagined and other systems repurposed (i.e., usually *Caco-2* cells have been cocultured to show interactions with macrophages [89]). For instance, one could try intact mucosal explants instead of *Caco-2* cells to more closely model the normal intestinal barrier [90]. Other underlying cancer cell types could also be tried [91]. However, the main finding here is that apical kale juice applied atop *Caco-2Bbe1* inserts still exerts anticancer. Kale juice emerged strongest transepithelially, dandelion juice was a close second, followed by green leaf lettuce, and spinach juice was last because of cytotoxicity. This rank order matched our original hypothesis, which came from the literature thus helping to validate our new coculture model system. That kale’s phytochemicals, themselves, actually cross the *Caco-2Bbe1* barrier seems evidenced by the neuroblastoma cells showing a signature MTT change (discussed later about MTT formazan dye reduction), well known for flavonoids [62,63,64].

Much hyperbole exists about kale as a superfood and anticancer [12]. This owes primarily to kale’s abundant types of phytochemicals. Direct experimental evidence for its juice being anticancer has, however, remained weak: extrapolated from dietary case-control studies of whole populations [2,3,4,5,6,7,8,9] and just one open-label trial in healthy people [37]. While a plethora of studies have documented the value of purified sulforaphane [92], resveratrol [34], and kaempferol [93], how these act in kale juice per se, has been questioned [7,13,18,35]. Some in vitro studies have used extracts of kale which are far different from what people actually ingest [11]. However, if we accept the correspondence of juices of cauliflower [42] and broccoli [41], then the list of kale juice susceptible cell types beyond *SH-SY5Y* might well include MCF7 and MDA-MB-23 breast cancer cells [42] and various adenocarcinomas and hepatic carcinomas [41]. By the same assumptions, many cell types are likely insensitive to the juices of *Brassica oleracea*, including *Caco-2Bbe1* shown herein. The others would include immortalized-but-not-malignant cell lines like NCTC-1469 [11], ECV304, VERO, Hep-2, MCF10A, and the 3T3 fibroblast cell line [42], none of which were effected by other *Brassica* juices. Most importantly, normal human liver cells were also not affected by a juice of *Brassica oleracea* origin [41]. A picture may therefore be emerging of cancer cell type selectivity for the juices of *Brassica oleracea*. Of course, such scattered in vitro studies must be verified by in vivo human studies.

According to a previous magnetic resonance imaging study of the adult human digestive tract [58], fluid volumes and transit dosages can be calculated after a human drinks a liquid similar to kale juice. From this we obtained the average volumes of stomach plus small intestines 1 h after a meal. From this, we calculated that the 4.2% (*v*/*v*) condition in our experiments is roughly equivalent to the luminal dilution after an adult drinks 42 mL of juice. Likewise, the 0.7% (*v*/*v*) condition is roughly equivalent to the luminal dilution after an adult drinks 7 mL of juice. With children, the amounts to drink to achieve the same would be proportionately less. On the other hand, digestive enzymes are known to diminish some phytochemical levels by as much as 3-fold according to one recent study [36]; in that case, our 4.2% dose would only be achieved by drinking 126 mL of the juice. That would not be too much for a meal because the previous clinical trial of kale juice required 200 g [37] (conversion form milliliters to grams under Methods).

How fast does kale juice act? In our monoculture studies, a single application of 4.2% kale juice showed initial anti-proliferative changes cells by 24 h (Table 1 and Figure 4). However, it took five days in open wells before all the neuroblastoma cells were dead (seen at either the low or high dose of kale juice: supplemental data with microphotographs). Perhaps more biologically relevant, it took three days for the anti-neuroblastoma activity of 4.2% kale juice to manifest transepithelially through the *Caco-2Bbe1*s in the co-culture model (Figure 2A). Perhaps to achieve the same in vivo effect where the food is moving would necessitate dosing several times a day.

Some comment is warranted about the rise in *Caco-2Bbe1* cell numbers seen in the co-cultures treated apically with either 4.2% kale or dandelion juice for three days (Figure 2B). Several factors maybe noteworthy. The first is this was not seen with low juice dosing (Figure 2B). Nor was it seen in open wells of immature *Caco-2Bbe1* cells at any dose of kale juice (Figure 4). For these reasons it did, at first, surprise. However, *Caco-2Bbe1* cells clearly change with time in culture. The cells only show the first mature characteristics by 11 days in culture [87]. Therefore, the state of these cells in our co-cultures between 15–21 days is distinct. Moreover, the finding in Figure 2B aligns nicely with higher TEER seen at 1 and 2 days after either high kale or high dandelion juice (Figure 1B). The findings also align with a previous report [94] showing that purple potato extracts promote the differentiation and TEER of *Caco-2Bbe1* cells [94]. The authors suggested that a purple potato diet could therefore be beneficial against leaky gut diseases [94]. If the same holds true for kale, then kale’s phytochemicals might not only cross the gut epithelium to selectively attack cancer cells, but also enhance the intestinal barrier. To explore this further, we studied the effect of adding *Lactobacillus* fermentum on TEER of *Caco-2Bbe1* cells (Figure 3). With the lactobacillus, we were able to dissociate a rise in TEER from inevitably causing lowered *SH-SY5Y* cell numbers in the co-cultures (Figure 3B), which is important and thought to be healthy.

How to interpret that kale was not totally unique? Not the only juice with transepithelial anticancer ability? With phytochemicals in all of them, each juice may be expected to exert some growth-stopping effects in line with earlier extract studies of lettuce [95], spinach [15], and dandelion [56,57]. Indeed, all three non-cruciferous juices showed degrees of anti-neuroblastoma if we waited long enough, 5 days (Figure S2). Spinach was actually quickest to act, but its overt cytotoxicity seems explainable by high level oxalate crystals [1]. A more subtle distinctive of kale came through when comparing the remaining two non-cruciferous juices, which seemed to act via pathways of apoptosis, while kale’s mode of action seemed through cell cycle arrest and cytostasis (Figure 2 and Figure S2). That is, kale juice alone induced high levels of ROS (Figure 5) and cell cycle arrest (Table 1).

The results with MTT have provided some confidence that at least some of kale’s flavonoids passed through the *Caco-2Bbe1* inserts to be taken-up by *SH-SY5Y* cells (Figure 3). Staining purple with MTT (Figure S1) is widely considered an index of the viability of mammalian cells, more specifically intracellular metabolic capacity and NAD(P)H flux [61]. Dead or slowly growing cell types are known to reduce little MTT, compared to rapidly dividing cells that exhibit high rates of MTT reduction [61]. This holds true except for a rare exception when taking up certain chemicals can also cause increased NAD(P)H flux and MTT dye reduction, even in the face of declining cell numbers [61]. Several plant antioxidant molecules act like this [63]. Bernhard et al. [62] first demonstrated this phenomenon using the CEM-C7H2 lymphocytic leukemia cell line during uptake of resveratrol which chemically reduced MTT molecules inside the cells in the face of declining cell growth. Similar findings have been documented repeatedly with plant extracts to the point where we now consider this MTT phenomenon a signature of such molecules [64]. Hence, when observed that basolateral *SH-SY5Y* cells in our co-cultures had declining cell numbers in the face of sustained high MTT metabolism (Figure 3), it most likely means that antioxidant flavonoids from kale juice had traversed the *Caco-2Bbe1* barrier and entered these cells. Of course, the precise molecules remain for more analytical chemistry than we performed, or if secondary effector signals were co-secreted during the process, remain topics of future study. However, this phenomenon in co-cultures allows us some assurance that a sizable amount of juice antioxidants got through and were taken-up.

Neurospheres are another important consideration in any *SH-SY5Y* cell studies (Figure S4). The formation of neurospheres is a sign of stressed neuroblastoma cells [55]. We observed no change in neurosphere numbers or sizes following kale or dandelion juice treatments (Figure S4). By comparison, lettuce juice seemed to temporarily reveal more neurospheres (anecdotal, Figure S5). Not seeing neurospheres with kale or dandelion is important because the drop seen in cell numbers after giving these juices cannot be explained artifactually by “hiding” in spheroids difficult to count [55].

More work is required to identify the transepithelial anti-neuroblastoma molecule(s) in our experiments. There are a vast number of biologically active molecules in kale [7,68], and in our chemical studies so far, we’ve not yet cross-examined the chemicals in the filtrates in the same way that led to our current focus on kaempferol (Figure 8B). Before extolling kaempferol, let us also note a smaller fluorescence HPLC peak mostly in dandelion and kale (to some extent) eluting at ~9.3 min, which maybe a stilbene. This may be suggested by comparison to the chromatograms of Remsberg, C. M. et al. [96] and in another study by Nikhil, K. et al. [97] where a pterostilbene conjugated with isothiocyanate was shown a potent candidate molecule. We also have excitation and emission spectral data to consider other than observed in Figure 7, and Table 2 [83,98,99,100]. According to Diaz et al. [83], trans-resveratrol solubilizes well in a basic environment and possesses fluorescence excitation and emission (λ_ex/em_ ~342/459 nm) closely matching some of our data in Figure 7. The 4′-OH group of resveratrol shown in Scheme 1 with pKa > 8.0 [98], is essential to (E)-resveratrol activity such as cytogenetic, radical scavenging, antioxidant, or antiproliferative agent [83]. A second emission band at 560 nm in Figure 7D (mainly for lettuce) suggests this juice could contain cis-piceid along with other polyphenolic compounds as was reported by Poutaraud, A. et al. [99]. The absorption (λ_abs_ ~330 nm) and fluorescence spectral profiles (λ_ex/em_ ~350/460 nm) of the vegetable juices also bear close resemblance with isoflavones [70] and flavanols [100]. Plus, the absence of a signal -N=C=S at ~1322 cm^−1^ band in the FTIR spectra (Figure 8A,B) of kale and dandelion, compared to lettuce and spinach, suggests that the isothiocyanates (SFNs) in kale and dandelion exist in forms other than as phenyl, methyl, tert-butyl, and ∝-naphthyl isothiocyanate [72]. The prominent broad doublet near 2150 cm^−1^ also indicates that kale, dandelion, lettuce, and spinach contain isothiocyanates to varied extents. It adds up that many chemicals in/from kale juice may have combined effects. Table 3 lists the known properties of kaempferol that line up better than sulforaphane or stilbenes with the prominence of kale juice.

What advantages and/or obstacles might be foreseen if using kale juice for children with neuroblastoma? We know of no contraindications for kale with other medicines used to treat cancer. Plus, there should be little worry next to the weight loss and lack of appetite that accompanies cancer. These patients often prefer soft food and drinks [108]. Standard chemotherapeutic side effects do, however, add some difficulties with swallowing, digestion, dehydration, and constipation. Some dietary interventions are known to help (i.e., ketogenic diets), but citrus fruits, especially grapefruit, are contraindicated with most drugs due to interfering with liver cytochrome enzyme metabolism [109]. The only other known juice in the literature showing promise for neuroblastoma is Bergamot juice, but it is from a citrus fruit [25]. Furthermore, isotretinoin, a vitamin A analogue, is useful for treating neuroblastoma, but it is difficult to swallow alone [110]. We should also not forget that neuroblastoma occurs worldwide and most of the world has limited access to such pills. Hence, if kale juice/smoothies can be definitively proven to be helpful for neuroblastoma patients, they should be safe and not prohibitively expensive.

## 5. Conclusions

The importance of our paper lies in the new in vitro co-culture model system being able to accurately rank order the anticancer potency of four green juices according to prior anticancer literature, which, in so doing, makes it clearer that kale and/or dandelion juices should cross the gut epithelium and work against internal cancers. Curly kale juice seems the best in our model, the only cruciferous vegetable studied, followed by dandelion and green leaf lettuce. Last was spinach juice, which was already suspected of some cytotoxicity due to its high level of oxalate crystals. Beyond this, kale juice’s anticancer properties are noteworthy because they were: (1) selective against the *SH-SY5Y* neuroblastoma cell line compared to the *Caco-2Bbe1* cell line co-cultured, (2) potent at enough dilution to be applicable to humans drinking a modest amount of kale juice in vivo, and (3) of a distinctive mechanism of action showing ROS and cell cycle arrest compared to the cell death profile certainly caused by spinach juice. Future animal-based studies are needed beyond these in vitro efforts to confirm and better determine the mechanism of anticancer action of kale and dandelion juices. Furthermore, juice-based diet studies of humans will be essential. It is anticipated that the co-culture model described herein can be modified appropriately to screen against other types of cancer cells, as well as to examine if these juices can act as adjuncts or adjuvants with standard chemotherapeutics.

## Figures and Tables

**Table 1:** Propidium iodide-stained flow cytometric analysis of *SH-SY5Y* cells given different juices for 24 h. Low dose is 0.7% and high dose is 4.2% juice. Values are the means from different experiments on different days. * Determined to be statistically significant, *p* < 0.05 (*n* = 4; paired *t* test).

Treatment and Dose	% of Cells in S Phase Arrest (*n* = 2 per Dose)
PBS low/high	8.9/6.4 (AVG of 4 = 7.7)
Kale low/high	10.5/11.0 (AVG of 4 = 10.7 *)
Dandelion low/high	8.1/6.3 (AVG of 4 = 7.2)
Lettuce low/high	8.9/5.9 (AVG of 4 = 7.4)
Spinach low/high	8.5/- * (AVG of 2 = 8.5)

**Table 2:** Peaks with significant intensities in the Attenuated Total Reflection–Fourier Transform Infra-Red (ATR-FTIR) spectra of the vegetable juices (kale, dandelion, spinach, and lettuce).

Possible Vibrations	Vibrational Frequencies of Vegetable Juice (cm^−1^)			
	Kale	Dandelion	Spinach	Lettuce
Aromatic –C=C stretching –OH stretching symmetrical and asymmetrical stretching vibration for the carboxyl ion (COO–) indicating the existence of carboxylic acid, ester, or carbonyl groups	B	b	B	1616 m
Part of isothiocyanate signature, deformations and stretching of aromatic C=C vibration of the aromatic rings. Also, in =C–O–C group of flavonoids	1560 s	1580 m	1560 m	1513 m
–O–H in plane bending in polyphenols deformation vibration of the carbon-carbon bonds in the phenolic group	1393 vs	1393 s	1393 m	1393 m
C–O stretching of acid groups bending vibrations of –CH_3_ or –CH_2_ groups in carboxylic acid, with more of isothiocyanate signature (–N=C=S). Also, an oxalate signature (1310 cm^−1^).	1330 b,vw	1330 b,vw	1322 s, 1310 s	1322 s
stretching vibration of the aromatic rings and =C–O–C group of flavonoids C–N stretching	1053 s	1060 vs	1056 m	1056 s
Sulforaphane signature (assigned to the S=O bond), the prototypic isothiocyanate	1033 vs	1033 m	1033 s	1033 vs

s—strong, w—weak, vs—very strong, vw—very weak, m—medium, b—broad band.

**Table 3:** Summary of main candidate molecules in comparison to published findings.

Reported Status	Sulforaphane	Kaempferol	Resveratrol/Pterostilbene
Concentration greater in kale than other three vegetables	Yes [80]	Yes [101]	Levels not reported [101]
Concentration in green curly kale	608 mg/100 g [36] *	46.8 mg/100 g [101]	microgram range [102]
Bio-accessible during digestion	34.1% [36] * Moderate	98.6% [46] High	“Low” [18]
Transported across *Caco-2* inserts (P_app_, × 10^−6^ cm/s)	31.2 [88] (good)	5.9 [46] (acceptable)	11.9 [103] (moderate)
Effect on *Caco-2* monocultures	Inhibits [47]	None [46]	Inhibits [104]
Effect on neuroblastoma monocultures Growth	G2/M arrest [47] **, mainly cytotoxic [92]	S arrest, mainly cytostatic [105]	S arrest, mainly cytotoxic [106]
ROS Effect in neuroblastoma	No change [81]	Increased [107] ***	Concentration dependent ↑↓ [84]

* In microgreens [36]. ** In *Caco-2Bbe1* cells. *** In glioblastoma cells, the closest cell finding. ↑↓ Depending on the concentration the stilbenes are reported to increase or decrease ROS (Reactive oxygen species).

## Data Availability

The data presented in this study are available on request from the corresponding authors. The data from JP are not publicly available due to policies of the LabArchives Mississippi College Edition (LabArchives, LLC, San Marcos, CA, USA).

## Supplementary Materials

The following are available online at https://www.mdpi.com/2072-6643/13/2/488/s1. A supplementary document is available which contains Figures S1–S5.
