# Supplementary material for: Transepithelial Anti-Neuroblastoma Response to Kale among Four Vegetable Juices Using In Vitro Model Co-Culture System
Source: Nutrients. 2021 Feb 2;13(2):488. doi: 10.3390/nu13020488 (PMC7913023; doi:10.3390/nu13020488)
Supplement: Supplementary file 1 [file nutrients-13-00488-s001.pdf]

## SUPPLEMENTARY MATERIALS

### Caco-2 and SH-SY5Y Individual Responses to Kale Assessed by MTT Staining

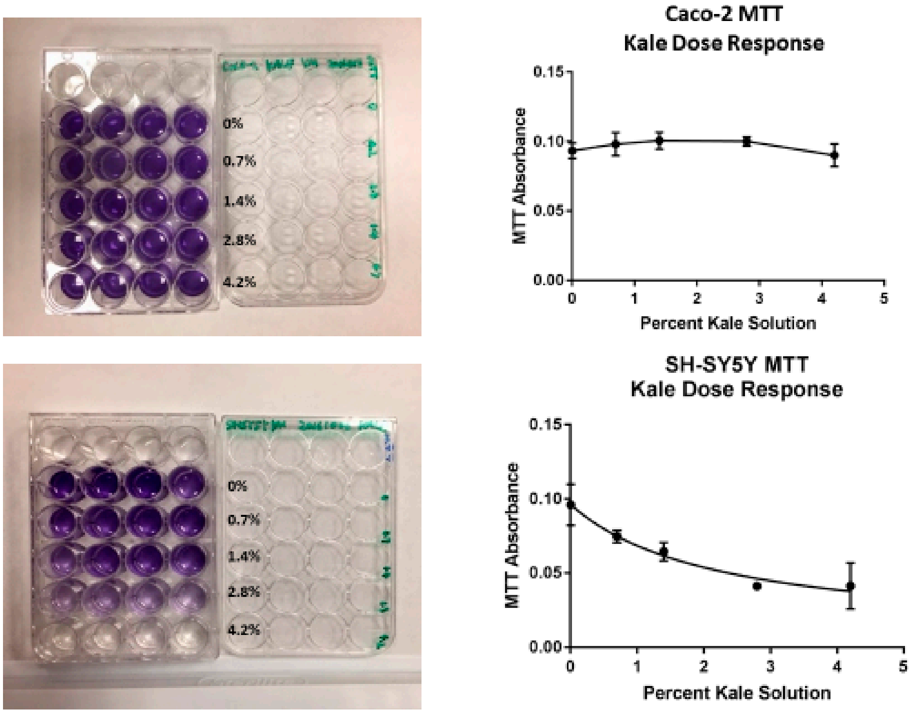

**Figure S1.** MTT metabolic profiles produced by open-well monocultures of Caco-2s (top panels) and SH-SY5Ys (bottom panels) in response to various doses of kale juice after 5 days. Both cell tyes were plated at 50,000 cells per well in 1 ml of complete media (10% FBS). Before receiving kale juice they were allowed to grow undisturbed into exponential growth phase by 4-days post-plating. The cells were then given media with reduced (1%) FBS media to match the 3D model set-up protocol, which favors that serum should not be in the lumen of the intesitine. After this, baseline cell numbers, diameters, and percent trypan blue staining were assessed before giving the juices. The remaining wells were replenished with 1 ml of complete media (10% FBS again) diluted with either PBS (4.2% vol/vol), or 0.7% - 4.2% kale juice (vol/vol). The top row (n=4 wells) received PBS, the second row (n=4 wells) received 0.7% kale juice, the third row (n=4 wells) received 1.4% kale juice, the fourth row (n=4 wells) received 2.8% kale juice, and the fifth row (n=4 wells) received 4.2% kale juice. Cells were washed in PBS before starting the experiment but to ensure we got all the cells, the floaters were added back. To the left are shown actual plates ending the MTT assay, showing MTT formazan dye changed color. Plots are to the right. Values represent mean ± SEM from three separata similar experiments. Our interpreation is that the kale juice acted only on the neuroblastoma cells. The Caco-2 curve shows no change with kale juice. With SH-SY5Y, the curve drops exponentially. The curve was drawn by GraphPad Prizm using a non-linear one-site exponential decay model.

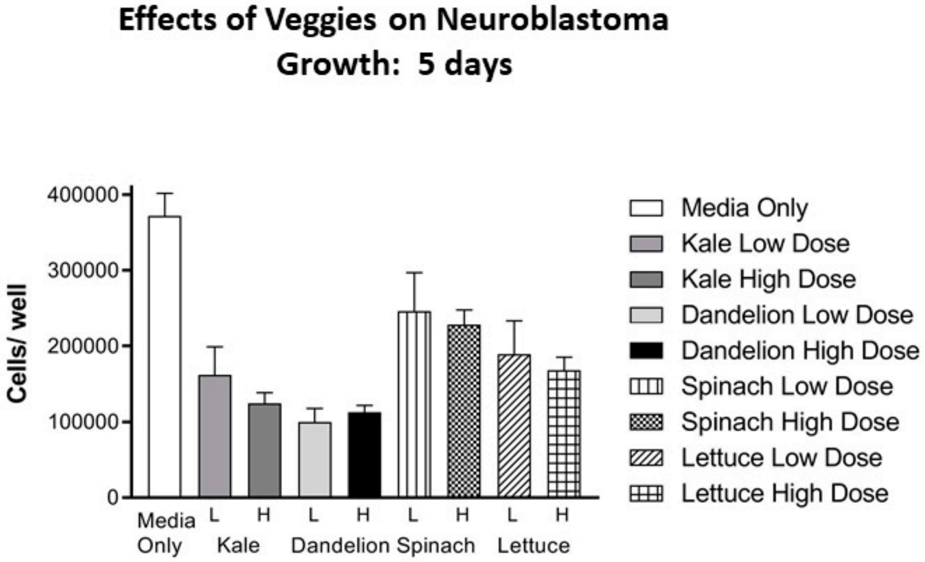


**Figure S2.** Effects of low (L: 0.7% vol/vol) and high (H: 4.2% vol/vol) dilutions of apical vegetable juices on SH-SY5Y cell numbers counted by cellometry (Nexelcom) in the 3D model after 5 days. The values come from pooled attached and floating cells, and represent total cells per well whether trypan blue excluding or not. However, the average percent viabilities (% trypan blue excluding) were as follows: SH-SY5Y from phosphate-buffered saline-high apically, 80.3% viability; SH-SY5Y from kale-low apically, 82.1% viability; SH-SY5Y from kale-high apically, 84.2% viability; SH-SY5Y from dandelion-low apically, 72.6% viability; SH-SY5Y from dandelion-high apically, 78.4% viability; SH-SY5Y from spinach-low apically, 70.1% viability; SH-SY5Y from spinach-high apically, 19.4% viability; SH-SY5Y from lettuce-low apically, 35.6% viability; SH-SY5Y from lettuce-high apically, 47.1% viability; Caco-2 from phosphate-buffered saline-high apically, 72.8% viability; Caco-2 from kale-low apically, 73.1% viability; Caco-2 from kale-high apically, 75.1% viability; Caco-2 from dandelion-low apically, 74.0% viability; Caco-2 from dandelion-high apically, 74.6% viability; Caco-2 from spinach-low apically, 72.5% viability; Caco-2 from spinach-high apically, 50.6% viability*; Caco-2 from lettuce-low apically, 71.4% viability; Caco-2 from lettuce-high apically, 75.5% viability. The data show almost no effects on the epithelial cell line. By comparison, after 5 days of apical kale juice in the 3D model (H or L), the SH-SY5Ys appear in a state of cytostasis (nondividing low numbers, yet high viabilities). This was unique from the other juices where lowered cell numbers instead match cytotoxicity (lowered neuron numbers plus lowered viabilities; particularly evident with lettuce and spinach). Asterisks mark statistical group differences from phosphate-buffered saline controls. Graph bars represent the mean ± SEM (n= 4-5 experiments per condition.

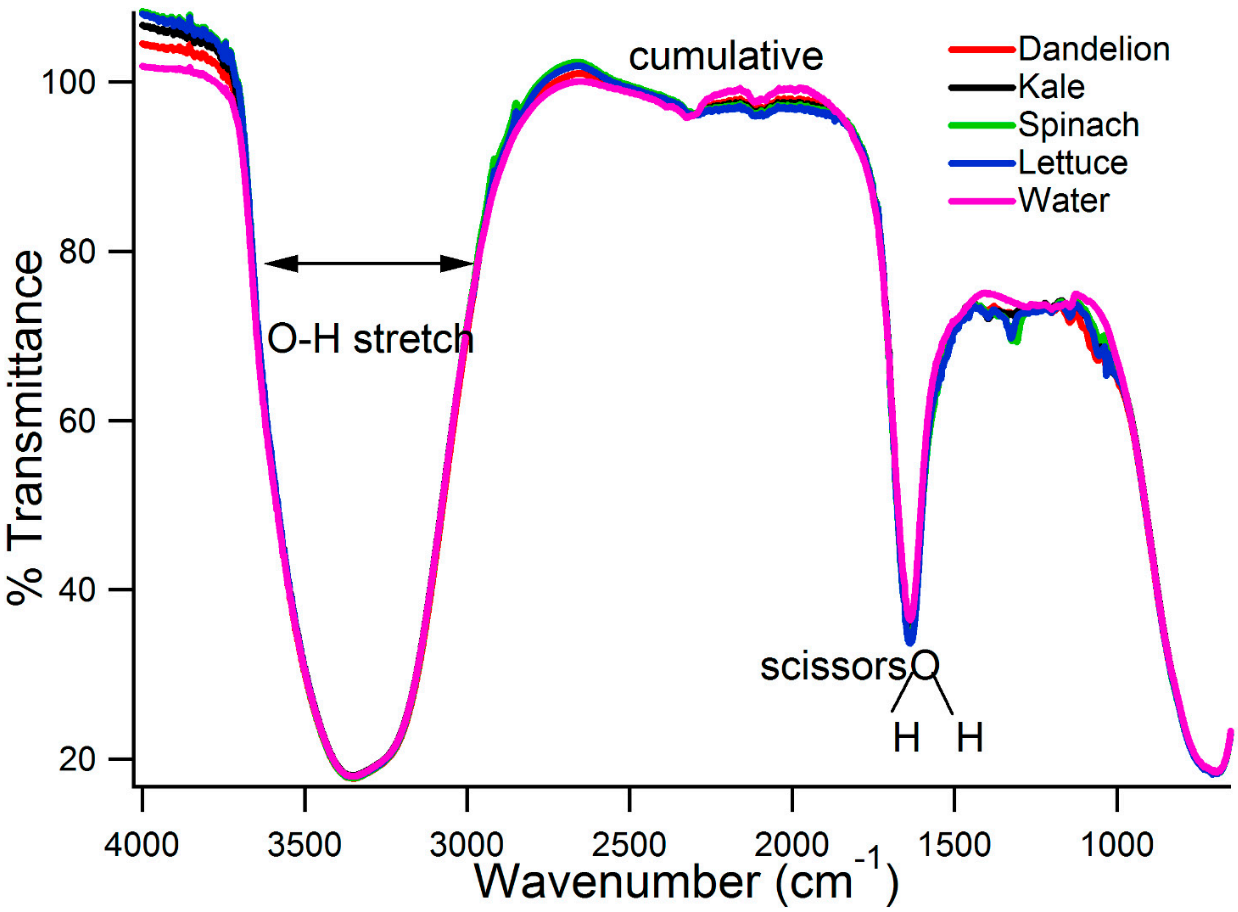


**Figure S3.** ATR-FTIR spectra of water extracts of leaves of kale (black), dandelion (red), spinach (green) and lettuce (blue) and water (pink) in the region 650-4000 $cm^{-1}$.

OPEN WELL TREATMENT WITH KALE JUICE (4.2%): NO NEUROSPHERE INDUCTION

| Starting state just after 24 hours in 1% FBS | 1 day after restoring 10% FBS +/- Kale Juice | 2 days after restoring 10% FBS +/- Kale Juice | 3 day after restoring 10% FBS +/- Kale Juice | 4 days after restoring 10% FBS +/- Kale Juice |
|---|---|---|---|---|
| No PBS yet | PBS (4.2%) | PBS (4.2%) | PBS (4.2%) | PBS (4.2%) |
| 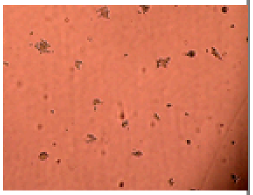 | 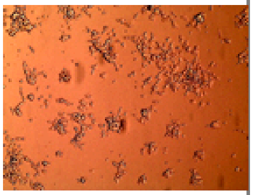 | 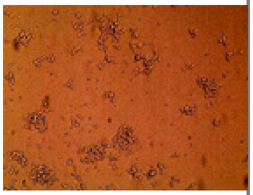 | 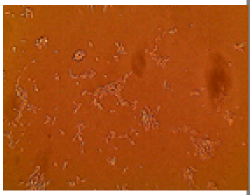 | 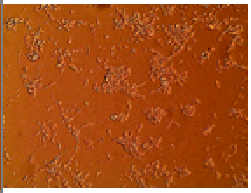 |
| No juice yet | Kale (4.2%) | Kale (4.2%) | Kale (4.2%) | Kale (4.2%) |
| Same as above | 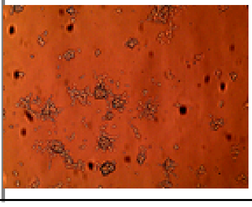 | 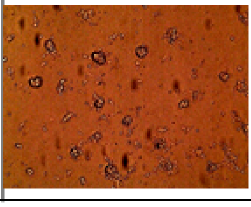 | 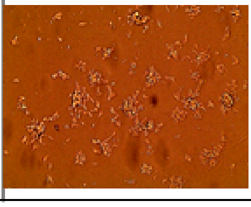 | 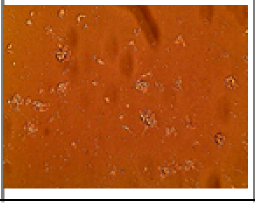 |

**Figure S4.** Phase contrast photomicrographs (10X objective) showing representative SH-SY5Y cells growing in open wells treated with kale juice (4.2%). The cells were plated at 50,000 per well in complete media four days prior to the first photos. After that, they were manipulated with different conditions the next two days as shown. The left most panels shows the neurons after 24 hours in media supplemented with just 1% FBS. This period of low serum was to mimic the 3D model conditions. That was replaced with complete media (10% FBS) diluted (vol/vol) with either kale juice (final 4.2%) or PBS (final 4.2%) and after 24 hours the second photomicrographs were taken. Additional photos go 4 days with same conditions. At this magnification, the purpose was daily monitoring of neuronal clumps and neurospheres (the large clumps that sometimes form).

## OPEN WELL TREATMENT WITH LETTUCE JUICE (4.2%): REVERSIBLE NEUROSPEHERES

| Starting state just after 24 hours in 1% FBS | 1 day after restoring 10% FBS +/- Lettuce Juice | 2 days after restoring 10% FBS +/- Lettuce Juice | 3 day after restoring 10% FBS +/- Lettuce Juice | 4 days after restoring 10% FBS +/- Lettuce Juice |
|---|---|---|---|---|
| No PBS yet | PBS (4.2%) | PBS (4.2%) | PBS (4.2%) | PBS (4.2%) |
| 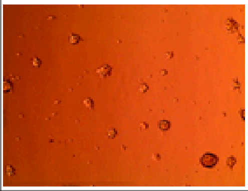 | 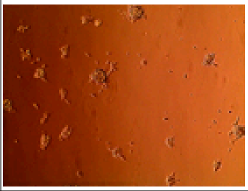 | 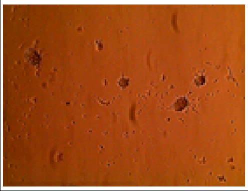 | 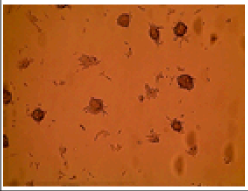 | 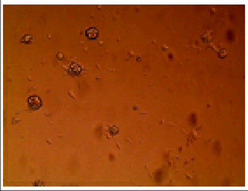 |
| No juice yet | Lettuce (4.2%) | Lettuce (4.2%) | Lettuce (4.2%) | Lettuce (4.2%) |
| 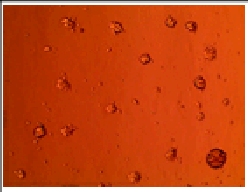 | 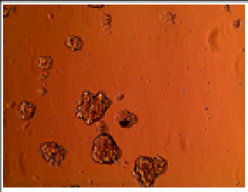 | 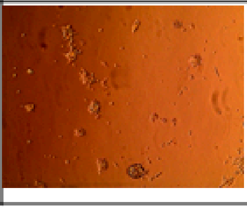 | 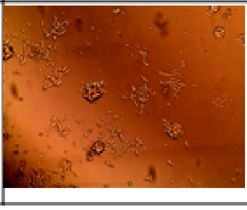 | 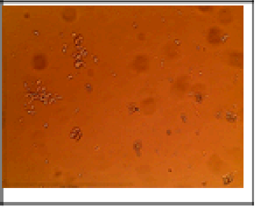 |

**Figure S5.** Phase contrast photomicrographs (10X objective) showing representative SH-SY5Y cells growing in open wells treated with lettuce juice (4.2%). The cells were plated at 50,000 per well in complete media four days prior to the first photos. After that, they were manipulated with different conditions the next two days as shown. The leftmost panels show cells after 24 hours in media supplemented with 1% FBS. That period in low serum was to mimic the 3D model conditions. That was replaced with complete media (10% FBS) diluted (vol/vol) with either lettuce juice (final 4.2%) or PBS (final 4.2%), and after 24 hours the second photos were taken. Additional photos were taken out to 4 days with the same conditions. At this magnification, the purpose was daily monitoring of neuronal clumps and neurospheres (the larger clumps that sometimes form).
